# Physiologically Based Pharmacokinetic Modeling of Efavirenz Nanoparticles: from Animal Model to Human Extrapolation

**DOI:** 10.1021/acsomega.5c05211

**Published:** 2025-09-23

**Authors:** Thalita Martins da Silva, Michelle Alvares Sarcinelli, Marcelo Henrique Cunha Chaves, Alan de Araújo Dias, Beatriz Ferreira de Carvalho Patricio, Livia Deris Prado, Leandro Tasso, Marcelo Dutra Duque, Helvécio V. A. Rocha

**Affiliations:** † Laboratory of Micro and Nanotechnology, Center for Technological Development in Health, 37903 FIOCRUZ, 4036 Brasil Avenue, 21040-361 Rio de Janeiro, Brazil; ‡ Postgraduate Program in Translational Research in Drugs and Medicines, Farmanguinhos, FIOCRUZ, 100 Sizenando Nabuco Street, 21041-000 Rio de Janeiro, Brazil; § Laboratory of Pharmaceutical and Technological Innovation, Department of Physiological Sciences, Biomedical Institute, UNIRIO, 94 Frei Caneca Street, 22290-240 Rio de Janeiro, Brazil; ∥ Laboratory of Analytical Development and Validation, Farmanguinhos, FIOCRUZ, 100 Sizenando Nabuco Street, 21041-000 Rio de Janeiro, Brazil; ⊥ Laboratory of Pharmacokinetics, Postgraduate Program in Health Sciences, 58802 University of Caxias do Sul, 1130 Francisco Getúlio Vargas Street, 95070-560 Caxias do Sul, Brazil; # Laboratory of Pharmacotechnic and Cosmetology, Department of Pharmaceutical Sciences, Institute of Environmental, Chemical and Pharmaceutical Sciences, UNIFESP, 210 São Nicolau Street, 09913-030 Diadema, Brazil

## Abstract

The present work aims to establish a formulation-specific, physiologically based pharmacokinetic (PBPK) model for efavirenz (EFV) nanocrystals that have shown increased dissolution and were produced following a top-down approach based on wet milling and spray drying by integrating solid-state characterization, in vitro performance, and preclinical pharmacokinetics to enable translational predictions in humans. The resulting material was thoroughly characterized using diffraction-based, spectroscopic, thermal, morphological, and particle sizing techniques, along within vitro dissolution testing and an in vivo pharmacokinetic analysis in rats. Then, a fully rat PBPK model was constructed using GastroPlus and incorporating the biopharmaceutical nanoparticle properties through the product particle size distribution (P-PSD) approach. The physiologically based biopharmaceutics model (PBBM) was validated with rat in vivo data and subsequently extrapolated to simulate human physiology. Compared with unprocessed EFV, nanocrystals exhibited superior dissolution efficiency (90.4% vs 52.6%) and a more homogeneous size distribution. Furthermore, the in vivo studies confirmed an increase in EFV exposure. The rat PBPK model accurately reproduced plasma profiles of both formulations, with all predictive error metrics falling within the acceptable 2-fold range. Extrapolation to human physiology revealed that a 350 mg EFV NC dose achieved systemic exposure comparable to that of standard 600 mg immediate-release tablet, but with faster absorption. Sensitivity analyses highlighted the critical influence of particle size and bile salt solubilization capacity on EFV oral absorption. This study pioneers the application of a fully mechanistic PBPK/PBBM model tailored to nanocrystal formulations of EFV. By bridging preclinical and human data through in silico simulation, the proposed approach supports dose optimization strategies and reinforces the role of nanotechnology in advancing nonbiological complex drug development.

## Graphical abstract

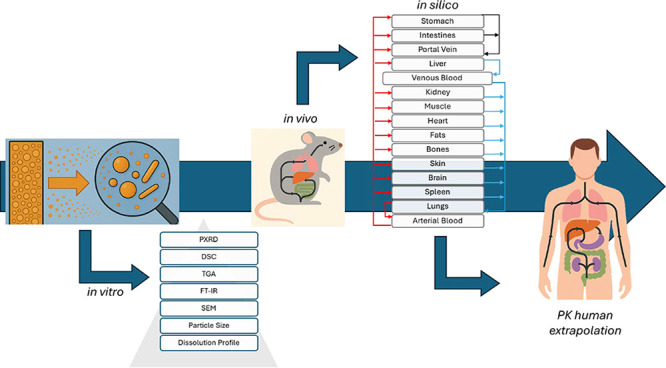

## 1 Introduction

The development of drug delivery systems is one of the main focus areas in the pharmaceutical sector.^1,2^ Although many of these systems were developed decades ago, they continue to be the subject of research objectives in the search for new medicines.^3^ Efavirenz (EFV) is a first-generation nonnucleoside reverse transcriptase inhibitor (NNRTI) used to treat human immunodeficiency virus (HIV) type 1 infection and to prevent the spread of HIV. Although its therapeutic use has decreased due to the emergence of new molecules, it is still indicated in the initial regimen in developing countries due to its efficacy and convenient dosage schedule.^4^ In addition, the drug has been studied for new uses such as pre-exposure prophylaxis (PrEP)^5^ or for repurposing in other diseases.^6,7^ Brazil has one of the most significant support programs for these patients worldwide, and EFV holds special symbolism in this country due to its compulsory licensing in 2008,^8^ the only case in the country to date. Furthermore, regardless of its clinical application, it is a drug that can be used as a model to evaluate the potential of new systems, given its biopharmaceutical properties, particularly its low aqueous solubility and dissolution problems.^9^

EFV is a crystalline “brick dust” drug, characterized by low oral absorption and bioavailability.^10–12^ It also has a low intrinsic dissolution rate, which is a limiting step for its in vivo absorption.^13^ These properties make EFV a suitable candidate for formulation strategies aimed at enhancing bioavailability. Some formulation approaches have been developed to improve the biopharmaceutical characteristics of EFV, including micelles,^12,14,15^ nanoemulsions,^16^ polymeric nanoparticles,^17,18^ solid lipid nanoparticles,^19,20^ or liposomes.^21,22^ However, many of these systems face limitations in terms of industrial scalability, and several studies did not show a real advantage in terms of bioavailability when evaluated through in vivo or in silico studies. Nanocrystals offer a promising approach by increasing the drug particle surface area, which, according to the Noyes–Whitney principle, accelerates the dissolution rate. This rapid generation of dissolved drug is particularly relevant for compounds like EFV, where absorption is driven by passive diffusion. By increasing the concentration of dissolved EFV in the lumen, nanocrystal formulations are expected to enhance the concentration gradient across the intestinal membrane, promoting faster and more efficient absorption.^23^ Unlike other drug delivery systems, nanocrystals consist almost entirely of the active pharmaceutical ingredient (API) with a minimal use of excipients, thereby enhancing ease of processing and enabling efficient scale-up.^24^

Our group has been working with EFV to improve its dissolution rate and bioavailability, and various approaches have been undertaken. First, we demonstrated the relevance of adequate control of the micronization process to achieve improved dissolution results in 600 mg tablets used in adult patients.^25^ Subsequently, we demonstrated the potential of co-micronization to enhance dissolution compared to the commercial micronization process of the pure drug.^26^ We achieved a robust method for obtaining microparticles with increased bioavailability through wet milling and spray drying, which was superior to previous results.^27^ In addition, we initiated an approach to develop specific formulations for pediatric patients,^28^ following the international recommendation of the World Health Organization.^29^ Finally, we turned to the development of nanocrystals,^30^ evaluating the extent to which reducing particle size to the nanometric scale could result in a more significant increase in bioavailability. The previously used process followed a bottom-up approach, while industrially, a top-down process has shown more promise, as evidenced by commercially available nanocrystal-based medicines, such as Tricor, Emend, and Rapamune.^31^ Thus, the production of nanocrystals through bead milling was proposed, and the results are presented in this article.

In parallel, our group has been dedicated to employing differentiated dissolution methods and modulating the pharmacokinetic profile to obtain a model that estimates biological behavior from in vitro and in silico results.^11,32^ This approach is in alignment with the latest recommendations from regulatory agencies^33^ and is an important focus of the pharmaceutical industry globally. However, a significant gap remains, which is still under development and leaves ample room for innovation, specifically regarding the use of in silico tools for modeling and pharmacokinetic extrapolation of nanostructured systems, which remain challenging due to their complex behavior.^17^

In this scenario, physiologically based pharmacokinetic (PBPK) models provide a mechanistic framework for simulating drug absorption, distribution, metabolism, and excretion based on anatomical, physiological, and biochemical parameters. When biopharmaceutical properties, such as dissolution profiles or particle size distributions, are integrated into these models, they are termed physiologically based biopharmaceutics models (PBBMs). PBBM allows for a more comprehensive understanding of how drug product quality attributes interact with physiological conditions to influence in vivo performance.^34,35^ This modeling approach is particularly valuable for nanotechnology-based formulations, where factors such as particle size and surface characteristics, biodistribution, and interactions with biological barriers significantly influence in vivo behavior.^17,36^ The growing relevance of model-informed drug development (MIDD) approaches reinforces the importance of PBPK and PBBM in guiding formulation development, supporting regulatory decisions, and informing drug product design.^34,37^

Given the clinical relevance of EFV in special populations, such as pregnant or breastfeeding women and pediatric patients, and its metabolism influenced by CYP2B6 genetic polymorphisms, as well as the frequent coadministration with drugs used in the management of HIV-related comorbidities, several studies have applied PBPK modeling to address these complexities. Costa et al.^38^ evaluated EFV pharmacokinetics during pregnancy, highlighting the need for dose adjustments based on gestational changes, sex, and CYP2B6 genotypes. Pan and Rowland Yeo^39^ investigated EFV exposure across mothers, children, and breastfeeding infants, supporting genotype-guided dosing in pediatric and perinatal care. Roberts et al.^40^ employed PBPK modeling to assess drug–drug interactions between EFV and levonorgestrel implants, suggesting dose adjustment strategies for women on antiretroviral therapy. Marzolini et al.^41^ modeled EFV’s dual role as an inducer and inhibitor of CYP enzymes. Additionally, Mtshali and Jacobs^42^ compared developed PBPK models for HIV and tuberculosis therapies, underscoring their value in simulating pharmacokinetics and interactions in co-infected patients.

While these contributions demonstrate the relevance of PBPK models for clinical decision-making, they predominantly focus on systemic pharmacokinetics. The present work aims to establish a formulation-specific, mechanistic pharmacokinetic model for EFV nanocrystals, which have demonstrated increased dissolution and were produced following a top-down approach based on wet milling and spray drying by integrating solid-state characterization, in vitro performance, and preclinical pharmacokinetics to enable translational predictions in humans. This work is one of the few studies using in silico models for nanocrystals that integrate biopharmaceutical data, and the first to apply a PBBM framework to simulate the formulation-specific in vivo performance of orally administered EFV nanocrystals.

## 2 Materials and Methods

### 2.1 Materials

All solvents used in this study were of chromatographic grade, and all reagents used were analytical grade. Sodium lauryl sulfate (SLS) was purchased from BASF (Ludwigshafen, Germany), and hydroxypropyl methylcellulose (HPMC) was purchased from ShinEtsu (TYLOPUR 603, Wiesbaden, Germany). EFV was purchased from Nortec Química (Rio de Janeiro, Brazil).

### 2.2 Sample Preparation

EFV nanocrystals (NCs) were prepared following the protocol described by Silva et al.^43^ Briefly, EFV (10% w/w) was dispersed in 600 mL of an aqueous solution containing 2% (w/w) SLS and 5% (w/w) HPMC. The dispersion was obtained using an Ultra-Turrax T25 homogenizer (IKA Instruments, Germany) at 11,000 rpm for 3 min. This presuspension was then subjected to wet milling in an agitator bead mill (DeltaVita 300, Netzsch, Germany) with yttrium-stabilized zirconium oxide beads (500 μm), retained in the milling chamber by a 300 μm mesh. Milling was performed in recirculation mode at controlled temperatures (<25 °C) using a peristaltic pump and a cooling system (FL4003, Julabo, Germany). A rotor speed of 2000 rpm was applied and maintained for 90 min. The mill was run in recirculation mode by using a peristaltic pump speed of 50 rpm. After milling, NC suspensions were spray-dried using a Mini Spray Dryer B-290 (Buchi, Switzerland) under the following conditions: inlet temperature, 150 °C; feed rate, 9 mL/min; aspirator flow, 35 m^3^/h; and drying gas flow rate, 600 L/h. Figure 1 illustrates the sample preparation workflow.

**Figure 1:**
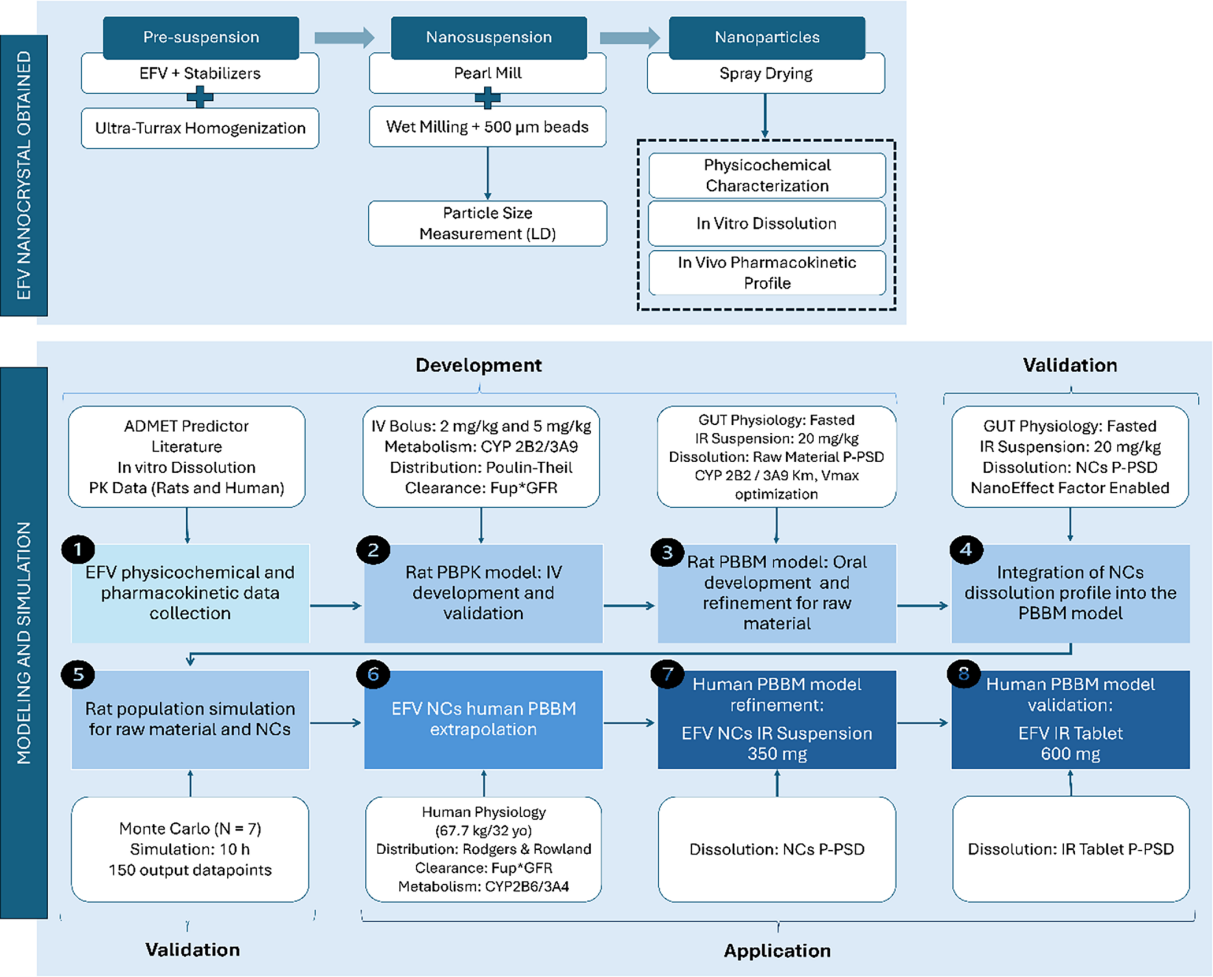
Workflow for preparation of EFV NCs and PBPK/PBBM modeling. Steps include NCs production, characterization, in vitro and in vivo evaluation, data collection, IV model development in rats, oral model refinement with NCs particle size data based on dissolution profile, population simulation, and human PBBM extrapolation.

### 2.3 Characterization of EFV Nanocrystals

#### 2.3.1 Powder X-Ray Diffraction (PXRD)

PXRD analysis was performed using an EMPYREAN diffractometer (Malvern Panalytical, Malvern, UK) equipped with a PIXcel3D-Medipix3 area detector. The measurements were conducted at room temperature using Cu-Kα radiation (λ = 1.541874 Å) with an operating voltage of 45 kV and a current of 40 mA. Data were collected with a step size of 0.01° over a 2θ range of 2°–50°. The crystalline structure of EFV was identified by comparing the experimental diffraction pattern with a simulated powder diffraction profile. The theoretical pattern was generated using Mercury 2024.3.1 (Build 428097), based on the crystallographic information file (CIF) retrieved from the Cambridge Crystallographic Data Centre (CCDC). The reference crystallographic structure was previously deposited in the CCDC database under deposition code 767883.^44^

#### 2.3.2 Fourier-Transform Infrared Spectroscopy (FT-IR)

Samples were analyzed using an infrared spectrometer (Nicolet iS50 FTIR, Thermo Scientific, USA) with OMNIC 7.0 software. Samples were analyzed in the ATR mode with a ZnSe crystal, scanning from 4000 to 400 cm^–1^ at 4 cm^–1^ resolution over 32 scans.

#### 2.3.3 Scanning Electron Microscopy (SEM)

Particle morphology was evaluated using a high-resolution field emission scanning electron microscope (FE-SEM, JSM-7100F, JEOL, Japan). SEM imaging was performed on spray-dried EFV NCs. Samples were deposited onto carbon adhesive tape fixed to an aluminum stub and dried at room temperature. After drying, the samples were coated with a thin layer of gold using a sputter coater (SCD 050, Bal-Tec, Liechtenstein) to improve conductivity. Imaging was performed at an accelerating voltage of 20 kV under high-vacuum conditions.

#### 2.3.4 Differential Scanning Calorimetry (DSC)

The thermal behavior of the samples was evaluated using a DSC TA 250 instrument (TA Instruments, USA). Analyses were conducted in hermetically sealed aluminum pans with a pinhole containing approximately 5 mg of each sample. Measurements were performed under a nitrogen flow of 50 mL/min with a heating rate of 10 °C/min, covering the temperature range from 30 to 200 °C.

#### 2.3.5 Thermogravimetric Analysis (TGA)

TGA and derivative thermogravimetric (DTG) analyses were performed using a TGA 5500 system (TA Instruments, USA). Experiments were performed from 30 to 600 °C, employing platinum pans containing approximately 5 mg of sample under a nitrogen flow of 50 mL/min. The heating rate was set at 10 °C/min.

#### 2.3.6 Particle Size Distribution (PSD)

The particle size distribution and surface charge of EFV formulations were assessed separately for the nanosuspension and spray-dried nanocrystal powder. Particle size distribution of the EFV nanosuspension was measured by laser diffraction (LD) using a Mastersizer 3000 (Malvern Instruments, UK) equipped with a Hydro MV wet dispersion unit, with purified water as the dispersant medium. During analysis, the dispersion unit was maintained under agitation (2700 rpm), and the sample was gradually introduced until 10% of obscuration was reached. Particle size was calculated based on Mie scattering theory,^45^ with refractive index parameters defined by the instrument software.

Additionally, dynamic light scattering (DLS) was performed using a Zetasizer Nano ZS90 (Malvern Instruments, UK) to determine the z-average diameter and polydispersity index (PDI). For this, 1 mL of each sample was diluted in deionized water and introduced into a folded capillary cell (DTS1070, Malvern, UK), with measurements taken at 25 °C and a detection angle of 90° using a He–Ne laser (λ = 633 nm). Under identical conditions, the zeta potential was also measured using the same instrument to evaluate surface charge and colloidal stability.

For the EFV NCs, particle size distribution was also measured using LD under conditions similar to those already described. Prior to analysis, the powder was reconstituted in the dispersant medium (0.01% (w/v) Tween 40 aqueous solution) to ensure proper redispersion and accurate measurement. The reconstitution procedure was carefully optimized to minimize agglomeration and preserve the particle integrity. The sample was sonicated for 1 min using the Mastersizer ultrasound, and the dispersion unit was maintained under agitation (2700 rpm). The sample was gradually introduced until optimal obscuration levels were achieved.

#### 2.3.7 In Vitro Dissolution Test

Powder dissolution studies were performed using a USP II apparatus (Evolution 6100, Distek, North Brunswick Township, USA). An amount equivalent to 100 mg of EFV was placed in vessels containing 900 mL of 0.25% (w/v) SLS solution, maintained at 37 ± 0.5 °C, with constant paddle stirring at 50 rpm. At specific time intervals (5, 10, 15, 30, 45, 60, 90, 120, and 150 min), 10 mL aliquots were collected and promptly filtered using a 0.1 μm poly­(vinylidene fluoride) (PVDF) membrane filter. After appropriate dilution, the EFV concentration was determined at 248 nm using a UV–vis spectrophotometer (Shimadzu, Kyoto, Japan). Both EFV NCs and the raw material were tested in triplicate. The dissolution profiles were analyzed in terms of dissolution efficiency, calculated using the trapezoidal method,^46^ and compared using the similarity factor (*f*_2_). The *f*_2_ calculations were conducted at the limit intervals of the dissolution plateau. When no plateau reached or the dissolution did not achieve 100%, a total of 85% of drug release was used.^47,48^ Data were calculated using the DDSolver, an add-in program for Microsoft Excel 365.^49^ An analytical curve was prepared with EFV concentrations ranging from 0.02 to 0.15 mg/mL. The NCs assay was determined as 90.32 ± 0.72% (data not shown).

### 2.4 In Vivo Studies

The pharmacokinetic (PK) study was performed following the methodology outlined by Prado et al.,^50^ which involved the PK evaluation of EFV formulations prepared as granules from EFV microcrystals. All procedures involving animals complied with the National Institutes of Health Guide for the Care and Use of Laboratory Animals and institutional ethical guidelines (protocol 006/2019, Universidade de Caxias do Sul). Male Wistar rats weighing 320–350 g were obtained from the Universidade Federal do Rio Grande do Sul (Porto Alegre, Brazil). Animals were housed in an air-conditioned facility under controlled conditions (23 ± 2 °C; 62 ± 3% relative humidity; 12 h light/dark cycle) with ad libitum access to food and water. They were acclimatized for 7 days before the study and fasted for 12 h before experimentation.

Two experimental groups were established: G1 (EFV, *n* = 6) and G2 (EFV NCs, *n* = 7). Both formulations, raw material (EFV) and nanocrystals (EFV NCs), were dispersed in a 1.0% aqueous carboxymethyl cellulose solution immediately prior to administration. Each group received a single oral dose of EFV (20 mg/kg) via oral gavage. A dosing volume of 1 mL was standardized across all groups to ensure consistency. Following dosing, blood samples were collected into heparinized tubes at predefined intervals (0.5, 1.0, 1.5, 2.0, 2.5, 3.0, 4.0, 6.0, 8.0, and 10.0 h). Plasma was isolated by centrifugation (12,000 rpm, 10 min; DTC16000, Daiki, Tokyo, Japan) and stored at −80 °C for subsequent analysis using a previously validated HPLC–MS/MS bioanalytical method.^51^ Pharmacokinetic parameters were estimated through Phoenix WinNonlin software, version 6.4 (Certara, Princeton, NJ, USA).

### 2.5 Computer Modeling and Simulation (M&S)

The development, validation, and extrapolation of the PBPK models for EFV were conducted using a combination of published and experimental data, with simulations performed in GastroPlus version 9.9 (Simulations Plus Inc., Lancaster, CA, USA) and its integrated modules: ADMET Predictor, PBPKPlus, Metabolism & Transporter, and Optimization. The modeling workflow (Figure 1) encompassed four main stages: (i) development and validation of a rat PBPK model, (ii) refinement with biopharmaceutical inputs to build a PBBM, (iii) application throughy simulation of EFV NCs performance, and (iv) extrapolation to human physiology.

PBPK modeling was employed in the initial steps to simulate EFV rat disposition based on its physicochemical properties and systemic pharmacokinetics using intravenous (i.v.) bolus data (Figure 1: steps 1 and 2). This provided a baseline for evaluating EFV metabolism, distribution, and clearance in the absence of absorption-related variability. PBBM modeling was then used to expand upon the PBPK structure by integrating the formulation-dependent biopharmaceuticals inputs, including dissolution data from the raw material to simulate the oral exposure (Figure 1: step 3), followed by incorporation of EFV NCs into the same framework (Figure 1: step 4). Finally, the model was extrapolated to human PBBM (Figure 1: steps 6–8) to assess the pharmacokinetic performance of the EFV NCs and compare the predicted parameters against clinical data from the reference IR Tablet.

Regarding preclinical studies, only single-dose studies were considered, ensuring that all animals were healthy, fasted, and received the drug by oral gavage. The administered doses ranged from 2 to 20 mg/kg, delivered either intravenously (i.v.) or orally (p.o.). Data were extracted using WebPlotDigitizer (Version 5.2, PLOTCON).

Modeling and simulation were performed using EFV physicochemical and biopharmaceutical properties retrieved from the literature or predicted based on its chemical structure obtained from the MolView v2.4.^52^ Table 1 summarizes the model’s input parameters.

**Table 1:** Physicochemical and Biopharmaceutical Properties of EFV Used in PBPK Models

	rat PBPK model	human PBPK model
parameter	value	value
molecular mass (g/mol)	315.67^a^	
diffusion coefficient (cm^2^/s × 10^–5^)	0.7473^a^	
Log *P*	4.6^a^	
p*K*_a_	10.2 ^53^	
solubilization factor	4342.8	
solubility (mg/mL)	0.009 (@pH 6.97)^54^	
solubility FaSSIF (pH 6.5)	0.182 ^32^	
solubility FeSSIF (pH 5.0)	0.847 ^32^	
Caco-2 *P*_app_ (cm/s × 10^–5^)	8.92 ^11^	
*P*_eff_ (cm/s × 10^–4^)	1.658	7.385
body weight (kg)	0.35	67.73 ^11^
B/Pr	0.92 ^54^	0.74 ^11^
*F*_up_ (%)	0.58 ^54^	0.22 ^11^
metabolism:		
CYP 2B2 *V*_max, PBPK_ (mg/s)	0.0517^b^	
CYP 2B2 *K*_m_ (mg/L)	0.9035^b^	
CYP 3A9 *V*_max, PBPK_ (mg/s)	0.0318^b^	
CYP 3A9 *V*_max, gut_ (mg/s)	0.00044^b^	
CYP 3A9 *K*_m_ (mg/L)	0.9278^b^	
CYP 2B6 *V*_max, PBPK_ (mg/s)		0.05^c^
CYP 2B6 K_m_ (mg/L)		3.914^c^
CYP 3A4 *V*_max, PBPK_ (mg/s)		0.00083^c^
CYP 3A4 *V*_max, gut_ (mg/s)		0.00044^c^
CYP 3A4 *K*_m_ (mg/L)		6.314^c^
*K*_p_ (per tissue)^a^:	Poulin–Theil	Rodger–Rowland
lung	7.52	0.65
adipose	69.58	27.59
muscle	3.66	3.09
liver	6.15	5.05
spleen	3.39	3.11
heart	5.06	1.97
brain	15.89	8.11
kidney	6.00	3.05
skin	8.44	3.79
reproductive organs	6.00	3.05
red marrow	8.09	8.86
yellow marrow	69.59	27.59
rest of body	3.39	3.11

a ADMET rredictor.

b Optimized from ref 55.

c Optimized from ref 56.

The simulated and observed PK data were compared using the fold error (FE) (eq 1). The predictive accuracy of the PBPK model for estimating observed concentration-time values in each single simulation was assessed by the average FE (AFE) and absolute AFE (AAFE) (eqs 2 and 3), as follows:

$$\mathrm{FE}=\frac{\mathrm{Pr}{\mathrm{edicted}}_{i}}{{\mathrm{Observed}}_{i}}$$ 1

$$\mathrm{AFE}=10^{1/n\times \sum \log(|\frac{\mathrm{Pr}{\mathrm{edicted}}_{i}}{{\mathrm{Observed}}_{i}}|)}$$ 2

$$\mathrm{AAFE}=10^{1/n\times \sum |\log(\frac{\mathrm{Pr}{\mathrm{edicted}}_{i}}{{\mathrm{Observed}}_{i}})|}$$ 3

where Predicted_*i*_ is the predicted concentration at time point *i*, Observed_*i*_ is the observed concentration at time point *i*, and *n* is the number of points at which the concentration was determined. The FE indicates the predictive accuracy of each data point, as shown in eq. 1. The AFE indicates whether the predicted profile underestimates or overestimates the observed values, as shown in eq. 2. The AAFE quantifies the absolute error from the observed values, as shown in eq. 3. If the FE of all the data points is between 0.2 and 2 (within 2-fold error) and the AFE and AAFE are both less than 2, it can be considered a successful simulation.^57^

#### 2.5.1 Rat PBPK Model Development

The following mechanistic assumptions were applied in the PBPK model: (1) EFV was absorbed from the gastrointestinal tract (GIT) via passive diffusion only,^58^ and (2) EFV was primarily metabolized by CYP2B6 and CYP3A4 in humans,^56^ with insignificant renal clearance.^54^ Based on these assumptions and considering the correlation between human and rat metabolism, hepatic clearance was modeled through CYP2B2 and CYP3A9.^59^ The rat *V*_max_ and *K*_m_ values^55^ were incorporated into the software to model the metabolism of these two enzymes, considering their expression in the PBPK model. For the initial simulations, an intravenous bolus administration was used as the dosage form.

A full PBPK model was developed based on the physiology of fasted healthy rats. Body weight was adjusted to 0.350 kg, and the Poulin–Theil (homogeneous) method^60^ was employed to estimate tissue partition coefficients (*K*_p_), assuming all tissues were perfusion-limited. Simulations were conducted with 2 mg/kg in a 1 mL solution. The renal clearance was defined based on the glomerular filtration rate scaled by the unbound fraction in plasma (*f*_up_*GFR).

The plasma concentration profile described by Balani et al.^10^ was used to optimize the *V*_max_ and *K*_m_ values and for fit and comparison purposes. The PBPK model was validated by simulating the administration of 5 mg/kg i.v.

#### 2.5.2 Rat PBPK Model Refinement and Validation

The model was further refined to account for oral administration. A mechanistic approach was adopted to integrate the dissolution data into the model and to evaluate the impact of particle size on EFV pharmacokinetics. The particle size distribution (PSD) of both EFV raw material and NCs was incorporated into the model’s database based on dissolution profiles in SLS 0.25% using the product PSD (P-PSD) approach.^61^ The P-PSD for both samples was calculated using the source data presented in Table 2. The resulting size distribution was incorporated into the PBPK model, with the mean particle size and standard deviation fitted across 10 bins. With this input, the model could then be considered a PBBM.

**Table 2:** Main Source Data for EFV P-PSD Calculation Based on In Vitro Dissolution Profiles^61^ ^,^ ^a^

parameter	value
true crystal density (g/mL)	1.395 ^44^
apparent (surface) solubility in aqueous phase at 37 °C (mg/mL)	0.009 ^54^
affinity for surfactant (K_aff_) (mg/mL/mM)^b^	0.0755 ^62^
concentration of surfactant in medium (mM)	8.7
size of micelle (nm)	1.26 ^63^

a All other parameters required for P-PSD calculations were maintained as standard data.

b Linear regression slope of EFV solubility as a function of SLS concentration in aqueous solution.

The parameters defined in the i.v. model were replied to simulate the oral administration of EFV raw material and NCs in rats. The GI physiology settings were defined to “Rat Physiological Fasted (Opt log *D* Model SA/V 6.1),” and the dosage form was defined as an immediate-release suspension (IR: suspension) to simulate the absorption of 20 mg/kg EFV administered by oral gavage over 10 h. Considering the dosage form, the stomach transit time was defined as 0.1 h, the standard value recommended for the single simulation mode.

Using the Optimization module in GastroPlus, CYP2B2 and CYP3A9 (*K*_m_ and *V*_max_) were adjusted to fit the pharmacokinetics profile of orally administered EFV, according to the data reported by Prado et al.^50^ Additionally, the solubilization ratio (SR) was fitted according to efavirenz solubility in biorelevant dissolution media and settled to 3.57 × 10^5^.

For nanocrystal pharmacokinetic simulations, additional biopharmaceutical considerations were included. The *Nano Effect* *Factor* was enabled in the dissolution model and set to 1.381, reflecting an enhancement in the apparent solubility and absorption efficiency for the smaller particles. The interfacial tension was estimated to be 0.02327 J/m^2^ based on the native solubility.

To account for physiological variability, Monte Carlo population simulations were performed (*n* = 7, 10 repeats) on the GastroPlus Population Estimates for Age-Related (PEAR) Physiology simulation option, generating 150 output data points for each virtual subject. The simulations were run over 10 h, and the probability contours were compared with the standard deviation observed in the in vivo studies. Both single and population simulations were conducted for 20 mg/kg oral EFV raw material and NCs suspension (IR).

#### 2.5.3 Rat PBBM Model Application: Extrapolation for Human Physiology

The rat model was extrapolated to human physiology to translate the rat-based PBBM of EFV NCs. A full human PBPK physiology was considered for a fasted healthy adult male (67.7 kg, 32 y.o.), using the anatomical and physiological parameters provided by GastroPlus. The tissue partition coefficients (*K*_p_) were calculated using the Rodgers & Rowland method (Table 1), and the renal clearance was defined based on *f*_up_*GFR, resulting in CL_renal_ = 7.12 L/h. The main metabolic enzymes CYP2B6 and CYP3A4 were modeled considering their expression in PBPK (2B6) and gut (2B6 and 3A4) (Table 1). The ACAT parameters were defined as a Human Physiological Fasted (Opt logD Model SA/V 6.1). As in the rat model, the same calculated nanocrystal P-PSD was incorporated into the drug record. A 350 mg dose of EFV NC suspension (IR) was simulated over 192 h.

By adjusting the PSD based on previously described dissolution profile of EFV 600 mg IR tablets in SDS 0.5%,^32^ the extrapolated human PBPK model was validated under the same developed conditions, simulating commercial EFV 600 mg IR tablet administration in the fasted state.

#### 2.5.4 Parameter Sensitivity Analysis

Parameter sensitivity analysis (PSA) was performed to assess the impact of biopharmaceutical and physiological properties on EFV absorption. The parameters investigated included EFV particle size, stomach transit time, and bile salt SR. The current, minimum and maximum values, as well as the number of tests are presented in Table 3. Logarithmic spacing was applied to all parameters.

**Table 3:** Parameter Settings Used for the Sensitivity Analysis of EFV Absorption According to the PPBM Model Developed

parameter	minimum value	baseline value	maximum value	number of tests
mean drug particle radius (μm)	1.14	11.4	114.0	25
particle radius SD (μm)	2	20	50	25
stomach transit time (h)	0.05	0.1	0.5	25
bile salt SR (cm/s)	3.5 × 10^4^	3.5 × 10^6^	3.5 × 10^8^	25

## 3 Results

### 3.1 Characterization of EFV Systems

#### 3.1.1 Powder X-Ray Diffraction

The PXRD patterns of the EFV raw material and NCs are presented in Figure 2. The analysis reveals a series of sharp and intense diffraction peaks characteristic of the bulk crystalline EFV. Even after processing, the material retains its crystalline state and polymorphic form, as evidenced by the presence of diffraction peaks at the same 2θ positions, which means that there is not a solid-phase transition. Although a new crystalline phase was not observed, the diffraction peaks appear less defined peaks and somewhat broadened, indicating a certain structural disorganization compared to the calculated powder pattern of EFV.^44^

**Figure 2:**
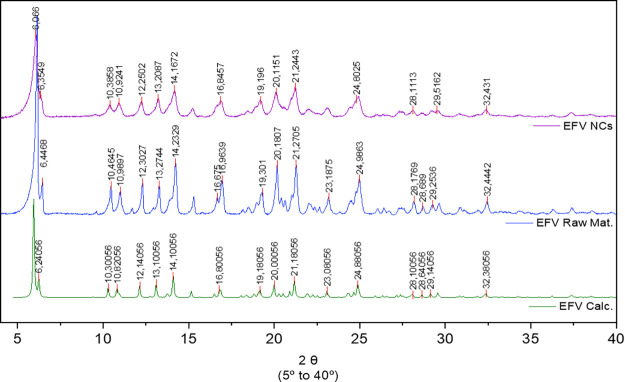
PXRD patterns of EFV raw material (blue) and NCs (purple) samples compared to the calculated one (green).

#### 3.1.2 Fourier-Transform Infrared Spectroscopy

The FTIR evaluation confirmed the chemical identity of EFV in both samples, with no significant shifts in characteristic bands, indicating the absence of phase transitions. These findings are consistent with the PXRD results. Given that EFV NCs are composed of a mixture of EFV, HPMC, and SLS, the ability of FTIR to detect band shifts associated with phase changes in the raw material is inherently limited by the complexity of the sample matrix.

Table 4 presents the band assignments for the samples, and the spectra are presented in Figure 3. The spectra were evaluated by comparing experimental and literature data to correctly attribute the vibrational modes.

**Table 4:** Assignment and Wavenumbers of IR Bands

	wavenumber/cm^–1^					
	–NH	–C≡C	–C=O	–C=C_(ar.)_	–CF	–C–Cl
Literature^64^	3315	2250	1747	1495	1186	1097
				1602	1198	
EFV API	3317	2249	1745	1495	1197	1096
				1602	1184	
EFV NCs	3315	2250	1742	1495	1195	1096
				1602	1182	

**Figure 3:**
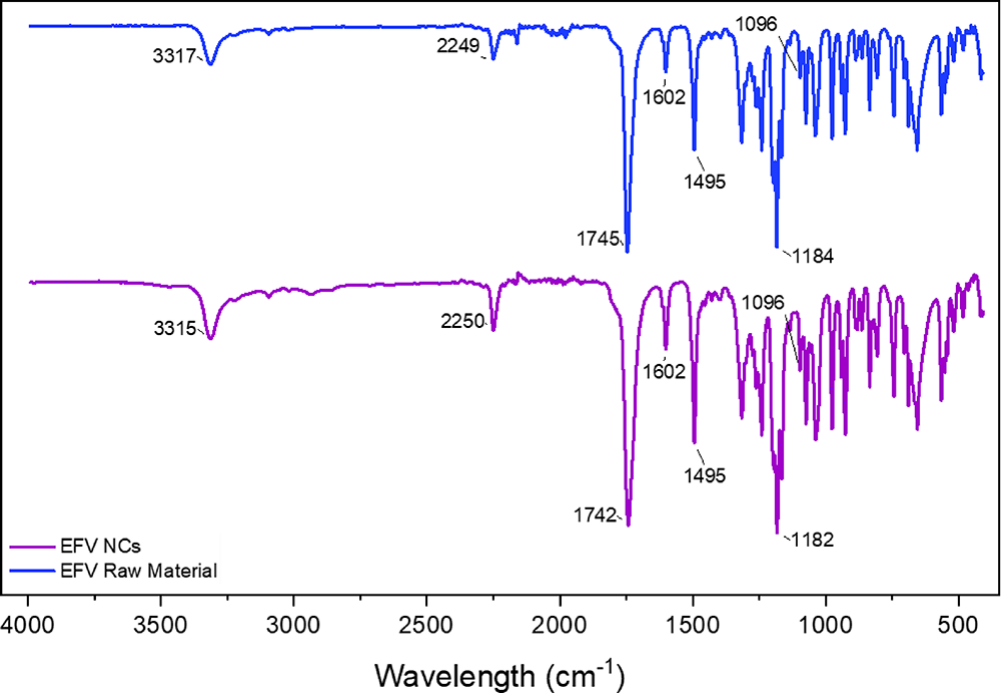
FT-IR spectra of EFV raw material (blue) and NCs (purple).

The −NH stretching region was observed at approximately 3315 cm^–1^ in both samples, with no significant shift after wet milling, supporting the chemical stability of EFV, and indicating that potential interactions with excipients are likely physical rather than covalent. A slight broadening and intensity reduction of this band were observed in EFV NCs, suggesting minor alterations in the local environment of the −NH group, which could be associated with physical interactions, such as hydrogen bonding, between EFV and the formulation excipients.^65^ The absorption bands corresponding to −C = O and −C≡C were identified at approximately 1747 and 2250 cm^–1^, respectively, with no differences detected between the samples. Similarly, the absorption bands associated with −C=C in the benzene ring, −CF, and −C–Cl also showed comparable results across both samples.

#### 3.1.3 Scanning Electron Microscopy

The SEM analysis provided clear evidence of the impact of the milling process on the morphology of EFV nanoparticles compared to unprocessed material. Figure 4 demonstrates that the spray-dried NCs exhibit greater uniformity and a smoother surface morphology than the raw material.

**Figure 4:**
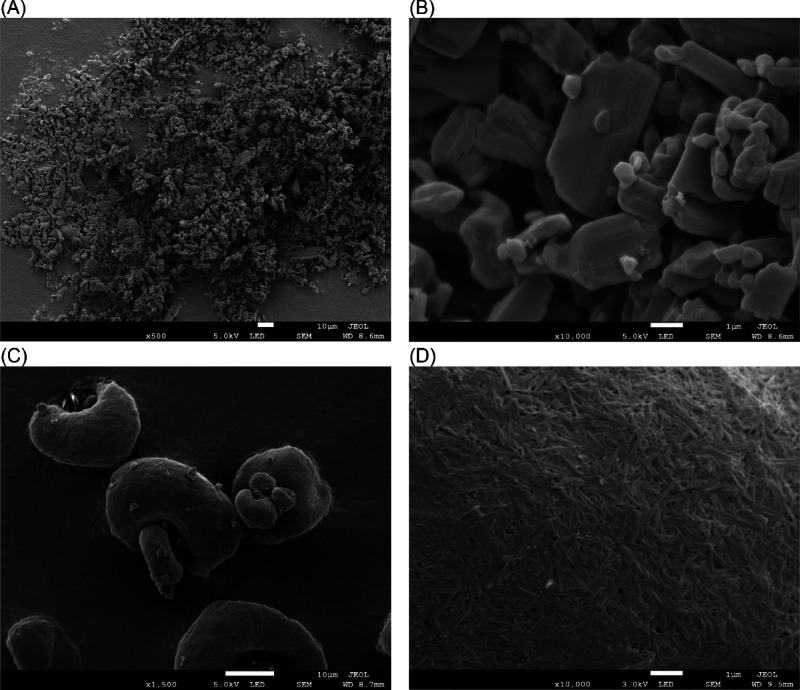
SEM imagens of EFV raw material (A, B) and NCs (C, D).

At low magnification, raw material particles (A) were characterized by large, densely packed aggregates with irregular shapes and sharp edges, reflecting their high tendency toward agglomeration.^25^ In contrast, NCs (C) formed more uniformly distributed clusters with a predominantly spherical morphology, highlighting the effectiveness of the milling process in achieving a more homogeneous distribution. At higher magnification, the raw material (B) particles revealed a rough surface with irregular structural features, whereas NCs (D) displayed elongated and structured forms, with a predominance of rod-like or fine shapes in a more uniform distribution.

#### 3.1.4 Thermal Analysis

The DSC profiles (Figure 5A,B), show that EFV raw materials exhibited a sharp endothermic peak related to its melting point (*T*_peak_ = 142 °C; *T*_onset_ = 136 °C). This endothermic peak was also found in EFV NCs, but with reduced enthalpy due to the presence of the excipients and possible changes in the degree of crystallinity. In the thermogram of EFV NCs (Figure 5B), a glass transition (*T*_midpoint_ = 105 °C) was also detected, suggesting that the high-energy milling process may have induced partial amorphization of the drug. Given the minimal amount of excipient, the thermal curves of EFV raw material and NCs curves show the same profile, evidencing that the crystalline structure was not fully altered.

**Figure 5:**
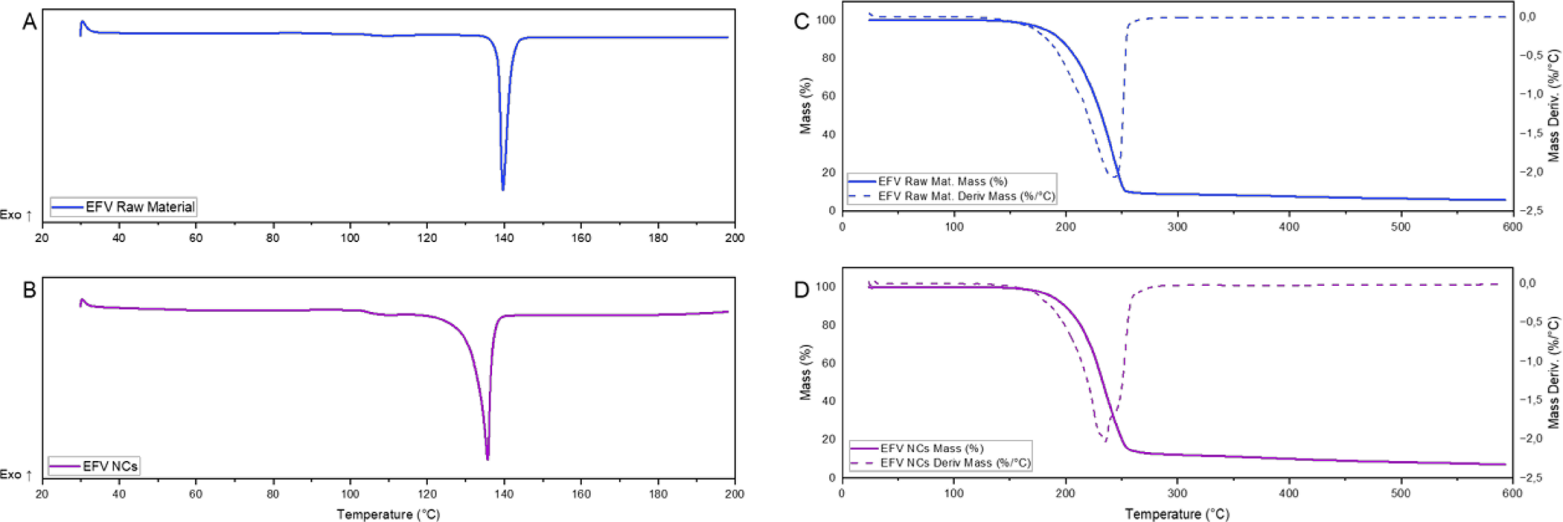
DSC (A, B) and TGA/DTG (C, D) thermograms of EFV raw material (blue) and NCs (purple).

To further investigate this behavior, the degree of crystallinity was estimated based on the enthalpy of fusion. The enthalpy of fusion for the EFV raw material was 51.31 J/g, corresponding to a crystallinity degree of 88.46% when considering the literature value of 58 J/g for pure crystalline EFV.^66^ For NCs, the measured enthalpy was 35.14 J/g. After correcting the EFV content in the formulation (90%), the enthalpy corresponds to 39.05 J/g, resulting in a crystallinity degree of 67.31%. These results support the hypothesis that although EFV NCs maintain their crystalline nature, the milling process induces a certain degree of structural disorder without leading to complete amorphization. This phenomenon was also reported by Silva et al.^43^ and Paiva et al.^67^ In the TGA curves (Figure 5C,D), a mass loss between 165 and 255 °C occurred in both cases, at temperature above the melting point of EFV (139–141 °C). This thermal event is characteristic of degradation, as previously reported.^28,68^

#### 3.1.5 Particle Size Distribution (PSD)

The particle size of EFV nanosuspensions was characterized using DLS and LD, two complementary techniques. Table 5 summarizes the results obtained after 30, 60, and 90 min of milling. The DLS measurements showed a progressive reduction in the *Z*-average particle size, from 30 to 90 min. The PDI remained below 0.2 throughout the milling period, with a slight increase observed after 90 min. The zeta potential decreased from −50.1 to −38.2 mV, indicating a gradual reduction in stability over time.

**Table 5:** Particle Size of EFV Samples as Suspensions after Milling and as Powders for EFV Raw Materials and Spray-Dried EFV NCs^a^

	suspension particle size							
	DLS			LD				
time (min)	*Z*-average (d.nm)	PDI	zeta potential (mV)	Dv_(10)_ (d.nm)	Dv_(50)_ (d.nm)	Dv_(90)_ (d.nm)	*D*_[3,2]_ (d.nm)	span
30	310.0	0.160	–50.1	133	300	661	250	1.761
60	277.7	0.166	–46.2	55	145	525	114	3.235
90	268.1	0.192	–38.2	42	108	452	86	3.785
	powder particle size (LD)							
				Dv_(10)_ (μm)	Dv_(50)_ (μm)	Dv_(90)_ (μm)	*D*_[3,2]_ (d.μm)	Span
EFV Raw Mat.				3.61	7.88	15.8	6.53	1.544
EFV NCs				8.57	20.2	35.7	13.2	1.342

a DLS – dynamic light scattering; LD – laser diffraction; EFV Raw Mat. – efavirenz raw material; EFV NCs – efavirenz nanocrystals.

After the first 30 min, the nanosuspension particles showed a lower PDI, signaling a narrow size distribution and reflecting the effective fragmentation of larger particles. At 60 min, the PDI remained within acceptable ranges, but the reduction in zeta potential and the increase in span indicated a slight heterogeneity.

Regarding LD measurements, as with DLS, after 30 min, the results indicated a relatively narrow size distribution. After 60 min, Dv_(50)_ decreased substantially to 145 nm, and Dv_(10)_ reached 55 nm, indicating a shift toward smaller and more dispersed particles. However, the increase in the span (3.235) suggests the onset of heterogeneity and the presence of larger particles or emerging agglomerates.

At 90 min, the distribution became more heterogeneous despite a continued reduction in Dv_(50)_. The Dv_(90)_ value decreased to 452 nm, yet the span rose further to 3.785, indicating a broad and asymmetric distribution. These results imply that although particle size reduction persisted, prolonged milling promoted particle reagglomeration, likely due to reduced electrostatic repulsion, as reflected in the lower zeta potential.

Although nanocrystals are expected to present a significant reduction in particle size, the spray-drying process led to mild aggregation, resulting in slightly higher Dv(50) values for EFV NCs (20.2 μm) compared to the unprocessed raw material (7.88 μm). As redispersion did not return the particles to the nanoscale in the dispersion media, DLS analysis was not performed on postdrying samples, and LD was selected as the most appropriate technique for characterizing the dried EFV NCs. To better understand the reconstitution performance, the surface-area-weighted mean diameter (*D*_[3,2]_), which is more sensitive to smaller particles, was used as a better estimate of the effective surface area available for dissolution. EFV NCs exhibited *D*_[3,2]_ of 13.2 μm, compared to 6.53 μm for the raw material. This apparent contradiction can be clarified by analyzing the dispersion profile of each formulation: while the raw material exhibited greater heterogeneity in size (span = 1.544), the EFV NCs PSD (span = 1.342) indicated better uniformity and polydispersity.

#### 3.1.6 In Vitro Dissolution Test

The NCs showed a higher drug release rate compared to the API. The samples achieved >85% dissolution within 15 min, and their release profile is a characteristic of very rapidly dissolving formulations (Figure 6B). It was evident from in vitro dissolution studies that the process used for particle size reduction of the raw material increased the dissolution of EFV. The dissolution efficiency (DE) was higher for the EFV NCs, with values of 90.36 ± 0.86% against 52.59 ± 1.00% for the raw material. The calculated *f*_2_ factor between the EFV API and NCs was 18.7, indicating that the profiles can be considered statistically different.

**Figure 6:**
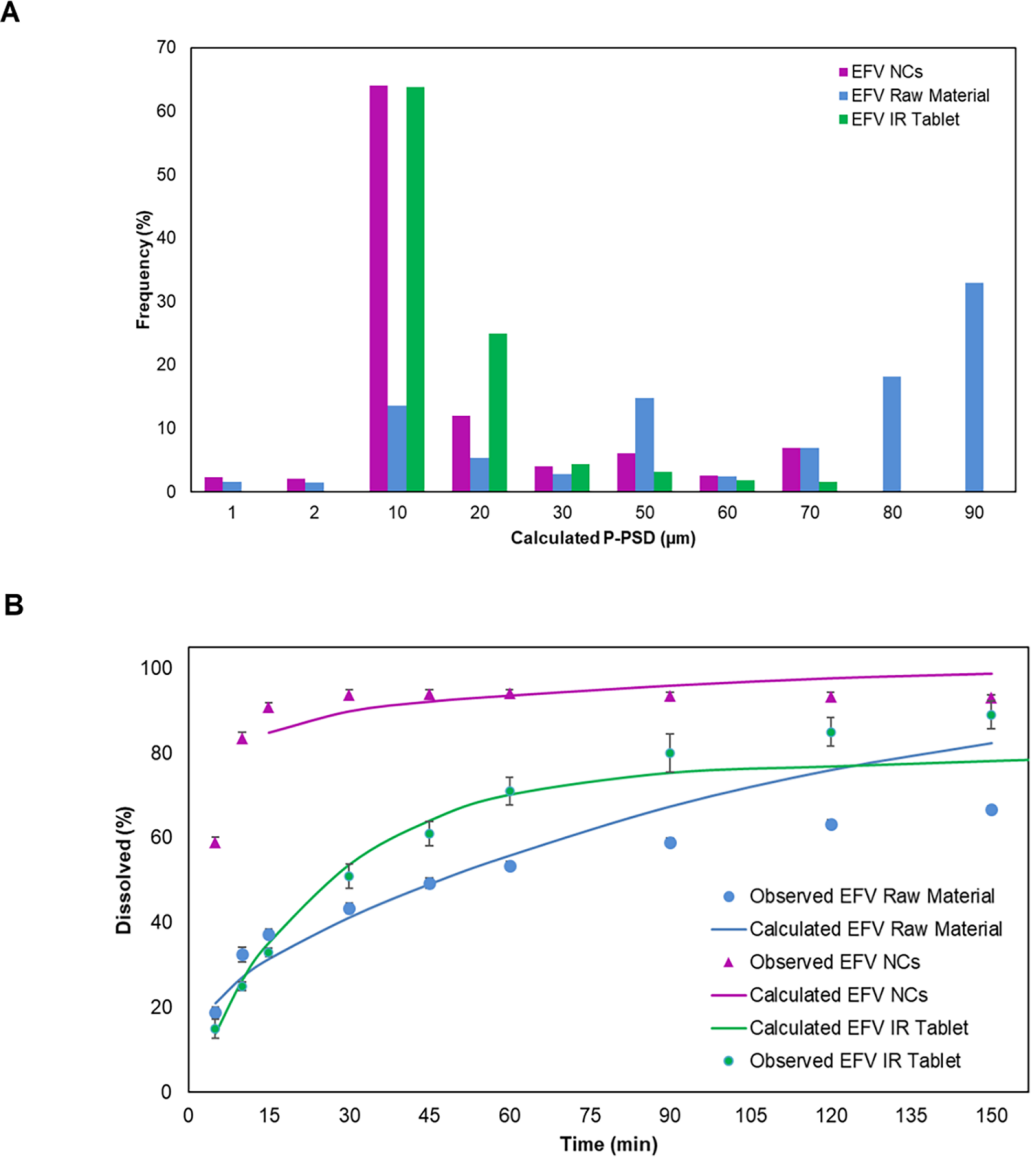
Calculated P-PSD for EFV raw material, NCs and IR Tablet (A), and its respective predicted and observed dissolution profiles in SLS 0.25% (raw material and NCs) and 0.5% (IR Tablet) (B). For observed dissolution values, data are presented as the mean and standard deviation. Data from IR tablet dissolution were previously published and adapted here with permission from da Silva et al.^32^ Copyright 2021 Taylor & Francis Ltd.

### 3.2 Computer Modeling and Simulation (M&S)

#### 3.2.1 Rat PBPK Model Development, Refinement, and Validation

The i.v. PBPK model successfully captured the distribution and elimination of EFV in rats. The predicted volume of distribution (*V*_d_) was 1.672 L. Figure 7 shows the overlay of simulated and observed EFV plasma concentration-time profiles, demonstrating a good fit between the predicted and experimental data. The correlation coefficients (*r* ^2^) for the predicted versus observed PK profiles were 0.93 for the 2 mg/kg dose and 0.88 for the 5 mg/kg dose. The model estimated AUC_inf_ values of 495.16 and 1239.2 ng·h/mL for intravenous doses of 2 and 5 mg/kg, respectively.

**Figure 7:**
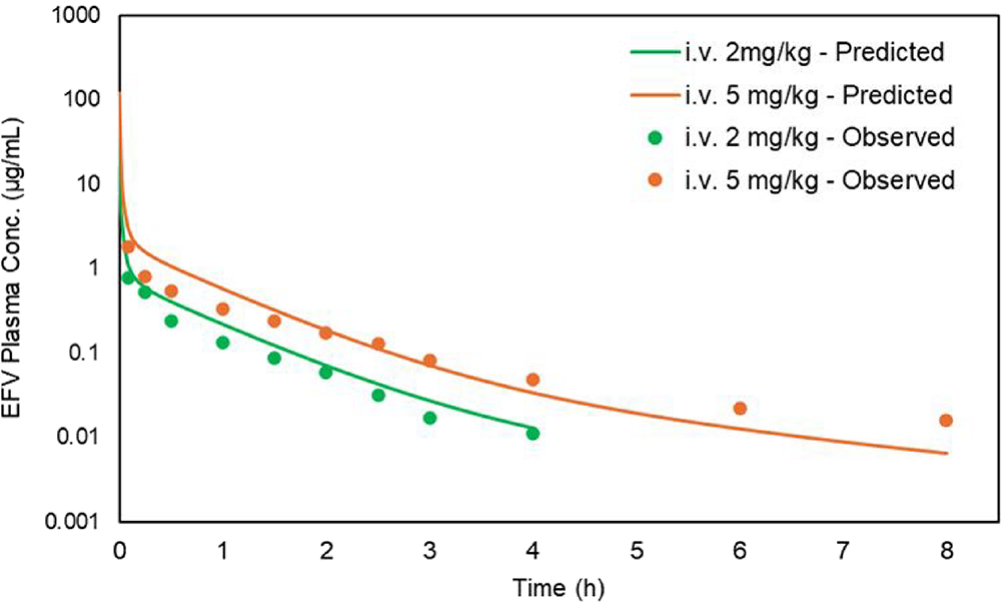
Predicted (lines) and observed (circles) plasma concentration-time profiles of EFV in rats (*n* = 3–5) after an i.v. administration of 2 (green) and 5 mg/kg (orange). Observed data were previously published and are adapted here with permission from Balani et al.^10^ Copyright 1999 Elsevier.

Subsequently, adjustments were made to the dosage form, dose, and GI physiology parameters to refine the predictive performance of the EFV oral PBPK model for rats. For metabolism, the kinetic constants for CYP2B2 and CYP3A9 were fitted according to the values described in Table 1.

Regarding the biopharmaceutical characteristics, the P-PSD for both samples and their respective calculated dissolution profiles are presented in Figure 6. The fitted distribution yielded a mean particle radius of 44.15 ± 42.5 μm (8 bins) and 11.4 ± 7.15 μm (10 bins) for EFV raw material and NCs, respectively. Additionally, the P-PSD for EFV 600 mg IR tablets was fitted to provide information for the extrapolated human model validation, resulting in a mean particle radius of 13.77 ± 27.5 μm (10 bins). The predictive performance of the P-PSD was supported by low FE values: for EFV raw material, AFE = 1.03 and AAFE = 1.13; for NCs, AFE = 1.02 and AAFE = 1.06; and for the IR tablet, AFE = 1.01 and AAFE = 1.05. After refinement, the single simulated PK profile for the raw material and NCs agreed with the observed in vivo data in rats (Figure 8A).

**Figure 8:**
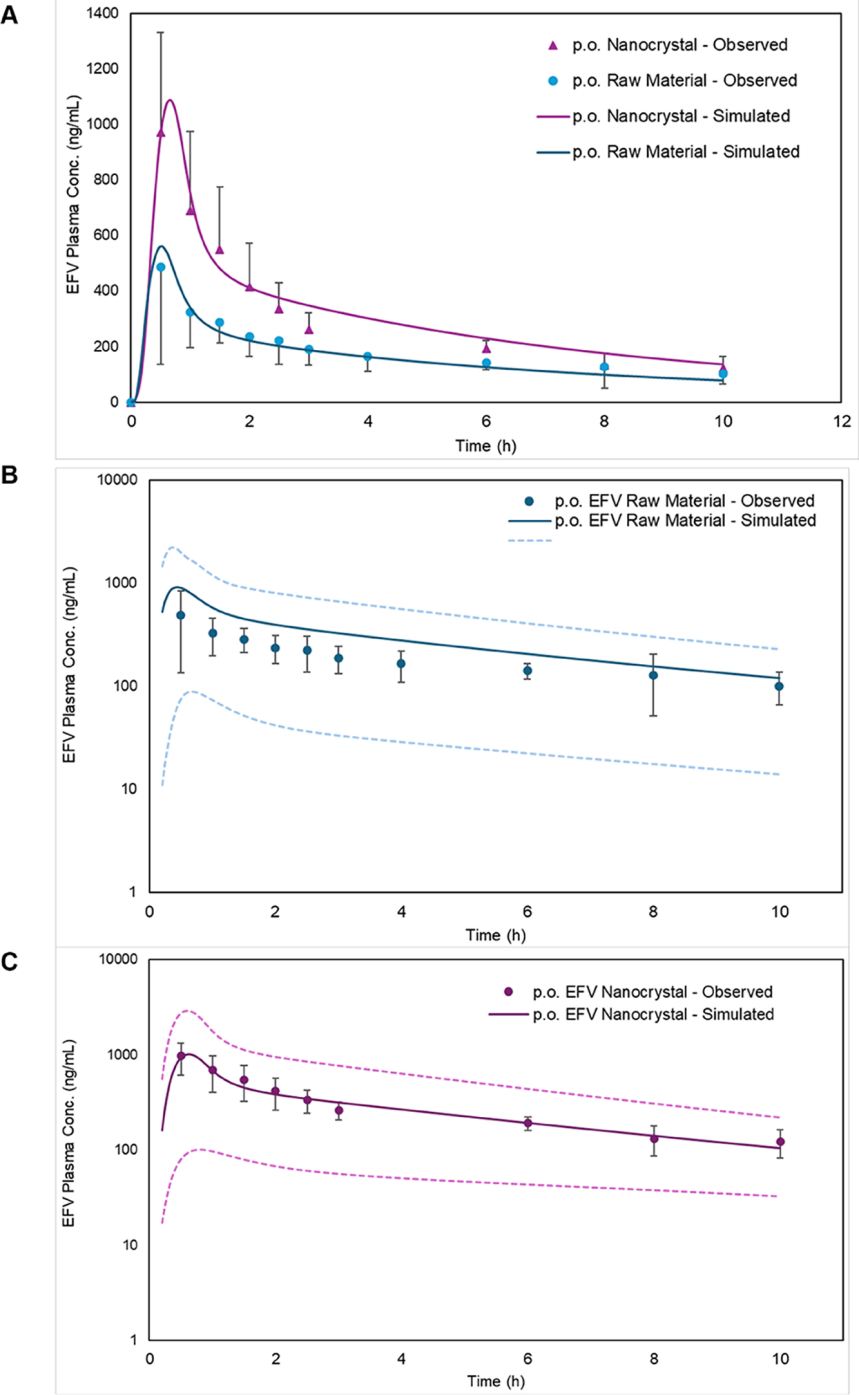
Pharmacokinetic plasma profiles of (A) observed and simulated profiles for EFV raw material (blue circles) and NCs (purple triangles) at 20 mg.kg^‑1^ p.o. in rats (*n* = 7). The population simulation for EFV raw material (B) and EFV NCs (C) was represented by the solid lines (blue for raw material and purple for NCs). The solid line represents the predicted mean; and the dashed lines represent the fifth and 95th percentiles of the predicted values for a virtual population. The observed data for EFV raw material was previously published and adapted here with permission from Prado et al.^50^ Copyright 2025 *Sociedade Brasileira de Química* (CC BY 4.0).

A population simulation was conducted for raw material and NCs administration in rats to account for physiological variability, and the results demonstrated that the plasma concentration-time profiles of the animals from the in vivo study were contained within the 95% prediction interval generated by the model (Figure 8B,C).

The predictive performance of the refined PBBM for both EFV raw material and NCs was evaluated by comparing simulated and observed PK parameters (Table 6). For the raw material, the model demonstrated good predictive accuracy, with an AFE of 1.02 and an AAFE of 1.05. Similarly, the NCs formulation model showed strong predictive performance, yielding an AFE of 1.13 and an AAFE of 1.13. All values fell within the commonly accepted 2-FE threshold,^57^ supporting the reliability of both models in adequately describing the pharmacokinetics of EFV in its distinct biopharmaceutical forms.

**Table 6:** Pharmacokinetic Parameters Predicted and Observed for EFV p.o. Administration of Raw Material and Nanocrystals in Rats

parameter	observed	simulated	FE	AFE	AAFE
raw material					
*C*_max_ (ng/mL)	521.2	560.4	1.07	1.02	1.05
AUC_t_ (ng.h/mL)	1739.6	1693.0	0.97		
AUC_inf_ (ng.h/mL)	3216.4	2368.8	0.74		
*t*_max_ (h)	0.58	0.50	1.00		
nanocrystals					
*C*_max_ (ng/mL)	970.4	1086.8	1.12	1.13	1.13
AUC_t_ (ng.h/mL)	2750.1	3120.6	1.11		
AUC_inf_ (ng.h/mL)	4016.1	4202.2	1.04		
*t*_max_ (h)	0.50	0.67	1.33		

#### 3.2.2 Rat PBBM Application: Extrapolation for Human Physiology

After developing PBBM in rats for EFV raw material administration and validating this model using the NCs pharmacokinetic data, the model was applied to human physiology by complete framework extrapolation. The primary hypothesis was that nanocrystal formulation would provide a faster absorption profile, as previously demonstrated in preclinical animal models.^43,67^

Some adjustments related to metabolism and distribution were implemented, based on experimental evidence and literature data, to ensure physiological relevance and improve the accuracy of the human PBBM. The extrapolated model reflected tissue-specific *K*_p_ values that align with the known high volume of distribution for EFV^69^ (*V*_ss_ = 746.6 L), particularly in lipophilic tissues such as adipose tissue and brain,^70^ supporting expectations of tissue accumulation consistent with the prolonged half-life (*t*_1/2_ = 72.7 h).^71^

Simulation of the plasma concentration-time profile following a 350 mg oral dose of EFV NCs yielded a *C*_max_ value of 2937.1 ng/mL, and an AUC_t_ value of 1.006 × 10^5^ ng.h/mL. Despite the reduced dose, the simulated *C*_max_ was comparable to the reference formulation^11^ (*C*_max_ = 2985 ng/mL; AUC_t_ = 1.38 × 10^5^ ng.h/mL), reflecting a faster and more efficient absorption of the nanocrystals and a shorter *t*_max_ (1.2 h vs 4.5 h). Notably, the terminal elimination phase of both observed EFV 600 mg IR tablet and simulated 350 mg NCs IR suspension PK profiles exhibited a parallel behavior (Figure 9A), indicating similar disposition kinetics after absorption. The *C*_max_ achieved with a lower dose supports the potential of nanocrystals for dose reduction strategies.

**Figure 9:**
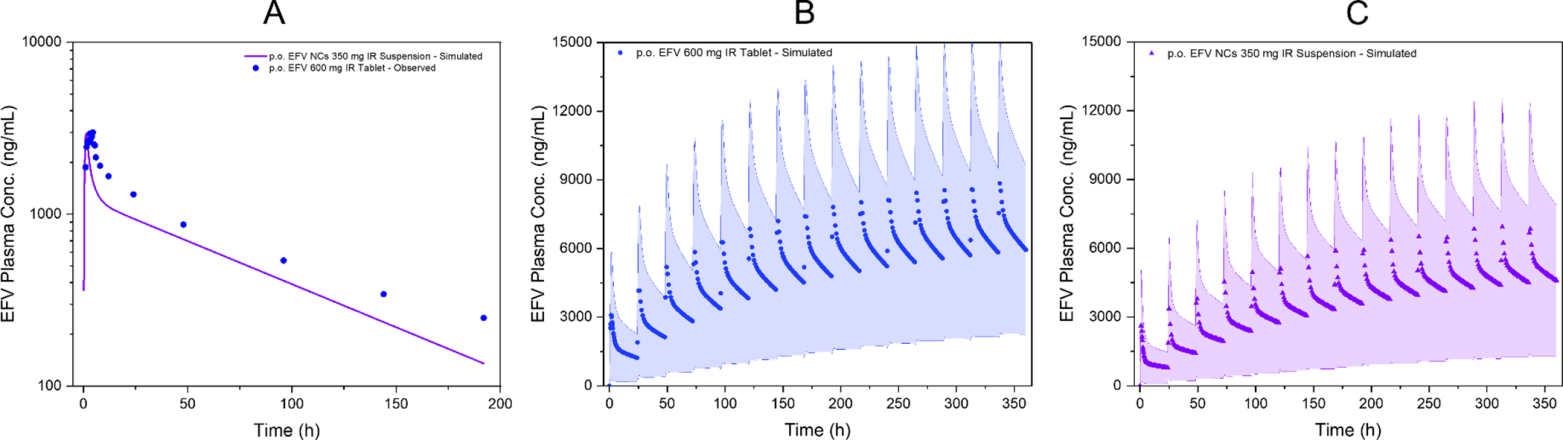
Human extrapolated EFV pharmacokinetic plasma profiles. (A) Presents a single simulation comparing observed EFV 600 mg IR Tablet (blue circles) and simulated 350 mg EFV nanocrystal IR suspension (purple line) in fasted humans. A population simulation (*n* = 18) of multiple doses of (B) 350 mg of EFV NCs (purple triangles) and (C) 600 mg of EFV IR Tablet, each day, for 15 days. The blue and pink area represents the 5th and 95th percentiles of the predicted values for the virtual population. Observed data in (A) were previously published and adapted here with permission from da Silva et al.^32^ Copyright 2021 Taylor & Francis Ltd.

The simulated pharmacokinetic profile for the p.o. administration of 350 mg EFV NCs was validated against published data corresponding to the administration of 600 mg EFV IR tablets, demonstrating satisfactory agreement with the observed mean concentration-time data (Table 7).

**Table 7:** Pharmacokinetic Parameters Predicted (Sim) and Observed (Obs) for Oral Administration of 350 mg EFV Nanocrystal IR Suspension and 600 mg EFV IR Tablets in Adults Using an Extrapolated PBBM Developed with Preclinical Data

	*C*_max_ (ng/mL)					AUC_t_ (ng.h/mL)				*t*_max_ (h)		
dosage form/dose^ref^	Sim	Obs	FE	PE_(%)_	Sim	Obs	FE	PE_(%)_	Sim	Obs	FE	PE_(%)_
nanocrystal IR 350 mg	3021.1				100,500				1.2			
IR Tablet 600 mg^72^	3327.5	4149	0.81	19.8	65,370	79,420	0.83	17.7	2.9	2.0	1.45	45.0
IR Tablet 600 mg^73^		2693	1.23	–23.6	51,020	54,475	0.93	6.34		3.65	0.80	20.5
IR Tablet 600 mg^11^		2930	1.13	–13.6	170,300	137,950	1.23	–23.4		4.5	0.65	35.6

Despite the lower dose, population simulations indicated similarity between the EFV NC formulation and the commercial IR tablet in pharmacokinetic parameters (Figure 9B,C). The predicted *C*_max,ss_ values were 8427 ng/mL for the IR tablet and 6462 ng/mL for the NCs, while AUC0−τ values reached 151,250 and 112,560 ng·h/mL, respectively. Importantly, both *C*_through_ (5567 ng/mL vs 4262 ng/mL) and average steady-state concentrations (*C*_avg_, 6302 ng/mL vs 4690 ng/mL) suggested that the nanosuspension achieves systemic exposure comparable to that of the IR tablet over the dosing interval. Additionally, the fluctuation index was acceptable for both formulations, with values of 45.4% for the IR tablet and 46.9% for the NCs, supporting a comparable pharmacokinetic profile under steady-state conditions.

#### 3.2.3 Parameter Sensitivity Analysis

The PSA demonstrated that bile salt SR and particle radius had the greatest influence on EFV absorption. As shown in Figure 10, increasing the bile salt SR resulted in a pronounced elevation of both absorption rate and extent. In contrast, longer stomach transit times and an increased PSD, marked by the standard deviation, negatively affected systemic exposure. Smaller particle radii were associated with higher *C*_max_ and AUC_t_, reinforcing the importance of particle size control in nanocrystal formulations.

**Figure 10:**
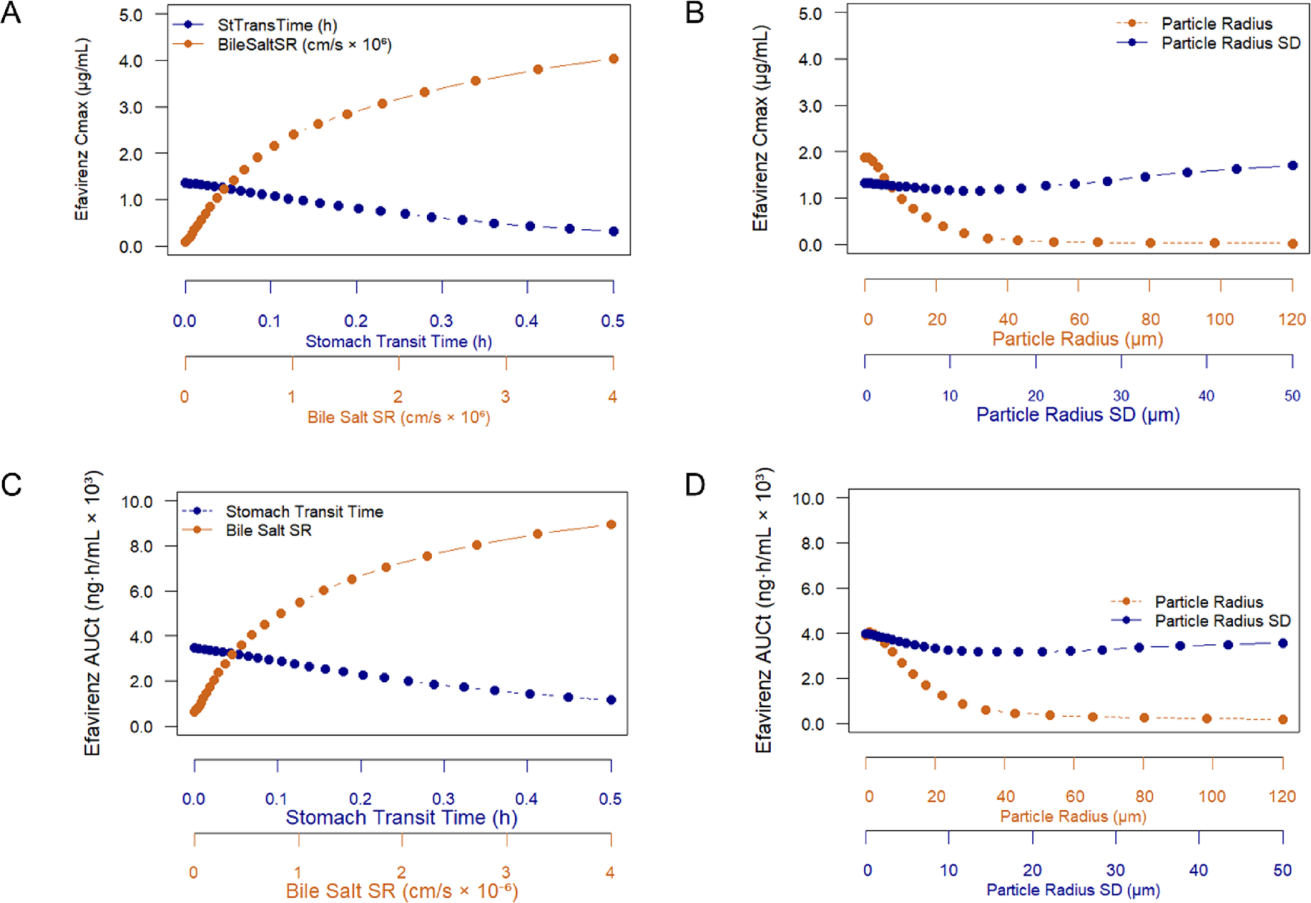
PSA for efavirenz absorption. (A, C) Impact of stomach transit time and bile salt solubilization ratio (SR) on *C*_max_ (A) and AUC_t_ (C). Impact of particle radius and particle radius standard deviation (SD) on *C*_max_ (B) and AUC_t_ (D).

## 4 Discussion

Over the years, our group has accumulated substantial expertise with the EFVI, as evidenced by a series of publications,^25,27,28,68,74^ as mentioned in the Introduction section. The methodology employed for preparing EFV nanocrystals was based on processes previously described by Silva et al.,^43^ who focused on the production of praziquantel nanocrystals, and Prado et al.,^50^ who explored the production of efavirenz microcrystals via milling.

The nanosizing process creates new interfaces and an increases Gibbs free energy, rendering nanosuspension formulations thermodynamically unstable. Therefore, particles tend to aggregate to minimize interfacial tension. This limitation can be mitigated using stabilizers, which enhance the wettability of hydrophobic drug particle surfaces and increase the activation energy required for aggregation. Two main stabilization mechanisms, electrostatic and steric, act as barriers to particle aggregation. Electrostatic stabilization is typically achieved with charged surfactants, whereas steric stabilization relies on nonionic surfactants or polymers. In this study, SLS was employed as an electrostatic stabilizer and HPMC, as steric stabilizer.^75,76^ Moreover, the presence of such stabilizers is essential not only during the wet milling process but also throughout drying, as they help mitigate aggregation. Here, spray drying was selected due to its scalability, process efficiency, and ability to produce powders that are more stable and compatible with solid dosage form development.

We have previously demonstrated that crystallite size can greatly impact EFV bioavailability.^68^ The PXRD pattern presented in Figure 2 suggests that the milling process may have induced a certain degree of disorder in the crystal particles. In case of complete amorphization, a characteristic halo would be detected in the analysis, as reported by Sun et al.^77^ This is an interesting result, since amorphization could induce some instability to the sample, in terms of both chemical degradation and recrystallization, potentially leading to the loss of dissolution enhancement afforded by the nanocrystal system. These findings closely correlate with the thermal analysis (DSC) (Figure 5), which revealed a broadening of the endothermic event, changes in fusion enthalpy and the appearance of a subtle glass transition (*T*_g_) in the EFV NC thermogram. The presence of *T*_g_ indicates that partial amorphization occurred during the milling process, as expected for high-energy processes. However, the presence of reflection peaks in the PXRD analysis of EFV NCs suggests that crystallinity was preserved to some extent.

The FTIR analysis confirmed that no chemical modifications were observed on EFV NCs, but the slight broadening and reduced intensity at ∼3315 cm^–1^ (Figure 3) indicated some minor changes in the local molecular environment and increased molecular mobility within disordered regions of the crystal structure. Feng et al.^65^ demonstrated that mechanical processing can induce crystal defects, which are distinct from amorphous regions, resulting in spectroscopic and thermal changes without complete loss of crystallinity.

Thus, besides particle size reduction, the disordering in the crystal structure revealed by PXRD, DSC, and FTIR, appears to be one of the factors contributing to the enhanced drug dissolution (Figure 7B). This findings reinforces the advantage of using multiple complementary techniques, providing a more comprehensive assessment and generating a database more relevant for interpreting the real characteristics of the system.

While the spray-dried NCs are expected to present a significant reduction in particle size, the LD measurements provide evidence that the process led to mild aggregation, resulting in higher Dv_(50)_ values for EFV NCs (20.2 μm) compared with the raw material (7.88 μm). Moreover, when examining the surface-area-weighted mean diameter (*D*_[3,2]_), the raw material showed a value of 6.53 μm, compared to 13.2 μm for the NCs. Nevertheless, the raw material exhibited greater size heterogeneity than the nanocrystals. Although some aggregation was observed after drying, the homogeneity of NCs is crucial to ensure a consistent dissolution behavior. The EFV NCs dissolution profile (Figure 6B) reflects the biopharmaceutical improvement achieved by the particle size reduction process, despite the incomplete return to nanoscale size evidenced by the LD measurements of the redispersed samples.

With respect to the dissolution method, an SLS 0.25% medium was applied to ensure sink conditions and allow a discriminative comparison between the in vitro performance of EFV NCs and the raw material. Although this dissolution medium is not physiologically representative of intestinal fluids, its use is supported by the literature, which demonstrates its discriminative capacity for EFV formulations. Sartori et al.^30^ reported that SLS 0.1% allowed an effective detection of performance differences among bottom-up EFV nanocrystals. Furthermore, da Silva et al.^32^ showed that dissolution in 0.5% SLS provided in vitro release profiles that were well correlated with in vivo absorption, comparable to results obtained with FeSSIF (Fed State Simulated Intestinal Fluid). These findings justify the use of SLS-based media as a practical and reliable surrogate for early-stage development.

The major integration between the physiological and biopharmaceutical characteristics of this work lies in the strategy adopted for incorporating dissolution data into the PBPK model through the P-PSD. This approach^61^ allowed a mechanistic integration of in vitro dissolution behavior directly into the absorption model, enhancing its predictive power and turns the PBPK model into a PBBM. Notably, the fitted mean particle radius for the EFV NCs was 11.4 ± 7.15 μm, whereas the Dv_(50)_ measured by laser diffraction for the same reconstituted powder was approximately 20.2 μm. This discrepancy is expected and reflects two key factors: (1) the modeling approach considers the effective surface area contributing to dissolution, not just the geometric size; and (2) the NCs redispersion capacity, reflected in a small mean particle size based on the dissolution profile.

When compared with the raw material, which exhibited a P-PSD fitted mean radius of 44.15 ± 42.5 μm, the NCs demonstrated a markedly smaller and more uniform size distribution. These characteristics were directly associated with the improved dissolution performance observed experimentally (Figure 7B). Importantly, reduced variability and improved control over PSD can lead to superior biopharmaceutical outcomes. Together, these results support the application of nanotechnology-based strategies to enhance the performance of poorly soluble compounds such as EFV.^25,78^

Several studies have demonstrated that EFV is not a substrate of key intestinal efflux transporters such as *P*-glycoprotein (*P*-gp) or breast cancer resistance protein (BCRP)^56,58^ and therefore no transporters were included in the EFV absorption model. EFV metabolism is attributed mainly to CYP2B6, with small contributions from 2A6, 1A2, 3A4, and 3A5 in humans.^56^ Here, we considered the major hepatic metabolism by CYP2B6 and some contribution of CYP3A4 expressed in the liver and intestines. As described by Martignoni et al.,^79^ the analogous isoforms CYP2B2 and 3A9 can be considered for EFV rat metabolism. Despite their structural similarity, these enzymes differ in terms of affinity (*K*_m_), catalytic efficiency (*V*_max_), and expression levels in both species. In the rat model, the optimized *K*_m_ for CYP3A9 was 0.9278 mg/L, whereas in humans, CYP3A4 exhibited a *K*_m_ of 6.314 mg/L. This difference highlights a substantially higher affinity of the rat isoform for EFV compared to the human enzyme, which was reflected in a greater contribution of intestinal extraction of EFV in rats, resulting in lower bioavailability than in humans (∼20% vs ∼60%).^10,71^ Such differences imply that under equivalent drug concentrations, EFV hepatic clearance in rats may reach saturation earlier than in humans, contributing to differences in clearance and systemic exposure across species. PBPK modeling could compensate for these differences through simultaneous consideration of enzyme abundance, intrinsic clearance, and physiological factors such as unbound fraction (*F*_up_). As shown in Table 1, both B/P_r_, and *F*_up_ present different values between rats and humans.

Moreover, different tissue partitioning models were adopted for rats (Poulin–Theil) and humans (Rodgers–Rowland) to better capture known differences in EFV distribution and elimination between species. EFV is known to accumulate in lipophilic tissues, such as adipose tissue and brain, contributing to its high *V*_d_ (>700 L) and prolonged *t*_1/2_ (∼72 h).^71^ The Poulin–Theil *K*_p_ calculation method was previously applied for predicting EFV distribution in rats brain tissue.^80^ However, compared with that method, the equations described by Rodgers–Rowland account, among others, for protein-binding and phospholipid interactions, allowing a more accurate prediction of the extensive EFV distribution.^81^

The PSA findings reinforced the critical role of particle size and solubilization capacity in modulating EFV absorption. Among all tested parameters, the bile salt SR and particle radius had the highest impact on EFV pharmacokinetics, supporting the relevance of controlling nanocrystal dimensions and dissolution-enhancing mechanisms. These results align with the P-PSD modeling approach adopted in this study and emphasize that, beyond average particle size, distribution homogeneity and biopharmaceutical interactions are key determinants of oral bioavailability for poorly soluble compounds such as EFV. Nanoparticles with controlled polydispersity have already demonstrated more consistent and predictable EFV release.

As for this study's findings, reflected in Figure 9 and Table 7, other studies^70,80,82,83^ have demonstrated that EFV distribution and elimination are not altered when absorption is complete, which supports the rationale for nanocrystals as a strategy for dose reduction. The refined PBBM model accurately predicted the PK profile of the EFV NC formulation, as reflected by low AFE and AAFE values. This greater precision may be attributed to the improved biopharmaceutical characterization of the nanocrystal system, underscoring the relevance of modeling tools integrated with full solid-state characterization for incorporating particle properties into pharmacokinetic predictions and evaluating the translational impact of novel delivery technologies.

In the contemporary therapeutic scenario, a group of drugs with increasing relevance is emerging, the nonbiological complex drugs (NBCDs). Unlike biological products, which are derived from living organisms, NBCDs are chemically synthesized but present structural and compositional complexity that challenges traditional approaches to the development and regulatory evaluation of conventional synthetic drugs.^84^ Nanocrystals have already been identified as one such complex system, as highlighted by Borchard.^85^ This complexity is relevant not only for new molecular entities (NMEs) but also for follow-on medicines, the so-called nanogenerics or nanosimilars.^37^ It is also noteworthy that highly relevant reports indicate that in vitro data do not always reliably predict the in vivo behavior, leading to failure in bioavailability tests.^86^ Therefore, it is essential to develop differentiated analytical methodologies capable of adequately characterizing the complexity of nanotechnology products.^87^ This includes techniques to measure the size, shape, surface charge, internal structure, and other relevant properties of nanoparticles.^88^ In the present case here demonstrated, the dissolution enhancement was followed by an increase in bioavailability, even at the preclinical stage.

Despite the successful prediction of EFV exposure using a full PBPK/PBBM model and its validation against both preclinical and clinical profiles, certain limitations must be acknowledged. The in vivo performance of the nanocrystal formulation has not yet been clinically evaluated. Moreover, some physiological conditions, such as the fed state or other comorbidities, may influence the absorption behavior in ways not fully captured by the model. Furthermore, while species-specific physiological and metabolic parameters were incorporated, the model does not account for interindividual variability in CYP2B6 expression, which affects EFV clearance and varies across populations due to genetic polymorphisms. Thus, although the simulations provide mechanistic support for the biopharmaceutical advantages of nanosizing and suggest the potential for dose reduction, confirmatory clinical studies remain essential.^89^

It is important to highlight that this kind of approach plays a critical role in generating robust and predictive results through in silico modeling and can enhance confidence in a go/non-go decision for subsequent clinical trials. The mechanistic IVIVE approaches in predicting the human PK of NBCD, such as EFV nanocrystals, by combining preclinical data, biopharmaceutical characterization, and species-specific physiological features within a PBPK framework, contribute to capturing formulation-dependent pharmacokinetic behavior, supporting rational decision-making in early clinical development.^17^ Interspecies differences are among the most critical challenges in translational medicine, and PBPK extrapolations can provide insightful for both NMEs and follow-on medicines.^90^ Here, PBPK models provided the systemic disposition framework, whereas PBBM models enabled mechanistic simulation for the oral administration based exclusively on formulation characteristics.

Taken together, these results highlight the translational applicability of the proposed modeling strategy. The extrapolated simulations suggest that a 350 mg dose of EFV NCs may achieve systemic exposure levels similar to those obtained with the commercial 600 mg IR tablet formulation, but with a short t_max_. From a clinical perspective, this finding supports the potential for dose reduction, which may help mitigate concentration-dependent adverse effects, particularly the central nervous system events often reported at higher EFV plasma levels.^91,92^ Additionally, lower doses may contribute to reduced production costs, an important consideration for public health programs.^93^ By incorporation of formulation-specific dissolution data into the model, this approach also provides a practical tool to support early development decisions and optimize formulation design before clinical evaluation.

## 5 Conclusions

The PBPK/PBBM modeling approach adopted in this study successfully predicted the pharmacokinetics of EFV nanocrystals in both rats and humans. The rat model served not only as a translational bridge but also as a validation strategy for assessing formulation impact. The in vivo study confirmed the improved exposure of EFV NCs compared with the raw material, which was well captured by the model using P-PSD derived from dissolution data . The validated rat model was then extrapolated to human physiology with adjustments in enzyme kinetics and tissue distribution models to reflect known interspecies differences.

To the best of our knowledge, this is the first study to describe the nanocrystal pharmacokinetics using a fully mechanistic PBPK model whiled reflecting the biopharmaceutical properties (PBBM) of the formulation under investigation. By integrating in vitro characterization data with preclinical pharmacokinetic outcomes, this model provides a formulation-specific approach that enhances the translational value of in silico simulations. The resulting framework contributes meaningfully to the rational development of nanotechnology-based drug products and supports future applications of PBPK modeling in the context of nonbiological complex drugs.

## References

[1] Khan M. I., Hossain M. I., Hossain M. K., Rubel M. H. K., Hossain K. M., Mahfuz A. M. U. B., Anik M. I. (2022). Recent Progress in Nanostructured
Smart Drug Delivery Systems for Cancer Therapy: A Review. ACS Appl. Bio Mater..

[2] Muhindo D., Elkanayati R., Srinivasan P., Repka M. A., Ashour E. A. (2023). Recent
Advances in the Applications of Additive Manufacturing (3D Printing)
in Drug Delivery: A Comprehensive Review. AAPS
PharmSciTech.

[3] Wu K., Kwon S. H., Zhou X., Fuller C., Wang X., Vadgama J., Wu Y. (2024). Overcoming Challenges in Small-Molecule
Drug Bioavailability: A Review of Key Factors and Approaches. Int. J. Mol. Sci..

[4] Costa B., Vale N. (2022). Efavirenz: History, Development and Future. Biomolecules.

[5] Duwal S., Seeler D., Dickinson L., Khoo S., von Kleist M. (2019). The Utility
of Efavirenz-Based Prophylaxis Against HIV Infection. A Systems Pharmacological
Analysis. Front. Pharmacol..

[6] van
Lier J. E. (2020). New Therapeutic Targets for Brain Function and Disease. J. Med. Chem..

[7] Chiou P.-T., Ohms S., Board P. G., Dahlstrom J. E., Rangasamy D., Casarotto M. G. (2021). Efavirenz
as a Potential Drug for
the Treatment of Triple-Negative Breast Cancers. Clin. Transl. Oncol..

[8] Brazil. Decreto N^o^ 6.108, de 04 de Maio de 2007. Concede Licenciamento Compulsório, Por Interesse Público, de Patentes Referentes Ao Efavirenz, Para Fins de Uso Público Não-Comercial; Diário Oficial da União: Brasília, 2007 (accessed May 25, 2025).

[9] Pawar J., Suryawanshi D., Moravkar K., Aware R., Shetty V., Maniruzzaman M., Amin P. (2018). Study the Influence of Formulation
Process Parameters on Solubility and Dissolution Enhancement of Efavirenz
Solid Solutions Prepared by Hot-Melt Extrusion: A QbD Methodology. Drug Deliv. Transl. Res..

[10] Balani S. K., Kauffman L. R., Deluna F. A., Lin J. H. (1999). Nonlinear Pharmacokinetics
of Efavirenz (DMP-266), a Potent HIV-1 Reverse Transcriptase Inhibitor. Rats and Monkeys. Drug Metab. Dispos..

[11] Honório T. d.
S., Pinto E. C., Rocha H. V. A., Esteves V. S. D., dos
Santos T. C., Castro H. C. R., Rodrigues C. R., de Sousa V. P., Cabral L. M. (2013). In Vitro–In Vivo Correlation
of Efavirenz Tablets Using GastroPlus. AAPS
PharmSciTech.

[12] Chiappetta D. A., Hocht C., Taira C., Sosnik A. (2011). Oral Pharmacokinetics
of the Anti-HIV Efavirenz Encapsulated within Polymeric Micelles. Biomaterials.

[13] Madhavi Bb, Kusum B., Krishna
Chatanya C., Madhu Mn, Sri Harsha V., Banji D. (2011). Dissolution Enhancement of Efavirenz by Solid Dispersion and PEGylation
Techniques. Int. J. Pharm. Investig..

[14] Maldonado S., Fuentes P., Bernabeu E., Bertera F., Opezzo J., Lagomarsino E., Lee H. J., Martínez Rodríguez F., Choi M. R., Salgueiro M. J., Damonte E. B., Höcht C., Moretton M. A., Sepúlveda C. S., Chiappetta D. A. (2025). Efavirenz
Repurposing Challenges: A Novel Nanomicelle-Based Antiviral Therapy
Against Mosquito-Borne Flaviviruses. Pharmaceutics.

[15] Fuentes P., Bernabeu E., Bertera F., Garces M., Oppezzo J., Zubillaga M., Evelson P., Jimena Salgueiro M., Moretton M. A., Höcht C., Chiappetta D. A. (2024). Dual Strategy
to Improve the Oral Bioavailability of Efavirenz Employing Nanomicelles
and Curcumin as a Bio-Enhancer. Int. J. Pharm..

[16] Mazonde P., Khamanga S. M. M., Walker R. B. (2020). Design,
Optimization, Manufacture
and Characterization of Efavirenz-Loaded Flaxseed Oil Nanoemulsions. Pharmaceutics.

[17] Ozbek O., Genc D. E., Ulgen O. K. (2024). Advances in Physiologically
Based
Pharmacokinetic (PBPK) Modeling of Nanomaterials. ACS Pharmacol. Transl. Sci..

[18] Amanda
Martinez L., Juliana Parente Menezes R., Jessica
Mendes N., Sinvaldo B., Traudi K., Andressa N., Paulo Vitor F. (2020). Efavirenz-Loaded Polymeric Nanocapsules: Formulation,
Development, and Validation of an RP-UHPLC-DAD Method for Drug Quantification,
Determination of Encapsulation Efficiency, Stability Study, and Dissolution
Profile. J. Appl. Pharm. Sci..

[19] Srivastava A., Gupta H. (2022). Preparation and Evaluation
of Efavirenz Loaded Solid Lipid Nanoparticle
for Improving Oral Bioavailability. Res. J.
Pharm. Technol..

[20] Mukubwa G. K., Safari J. B., Walker R. B., Krause R. W. M. (2022). Design, Manufacturing,
Characterization and Evaluation of Lipid Nanocapsules to Enhance the
Biopharmaceutical Properties of Efavirenz. Pharmaceutics.

[21] Okafor N. I., Nkanga C. I., Walker R. B., Noundou X. S., Krause R. W. M. (2020). Encapsulation
and Physicochemical Evaluation of Efavirenz in Liposomes. J. Pharm. Investig..

[22] Kenchappa V., Cao R., Venketaraman V., Betageri G. V. (2022). Liposomes as Carriers for the Delivery
of Efavirenz in Combination with Glutathione—An Approach to
Combat Opportunistic Infections. Appl. Sci..

[23] Babadi D., Dadashzadeh S., Osouli M., Abbasian Z., Daryabari M. S., Sadrai S., Haeri A. (2021). Biopharmaceutical and Pharmacokinetic
Aspects of Nanocarrier-Mediated Oral Delivery of Poorly Soluble Drugs. J. Drug Deliv. Sci. Technol..

[24] Magdum S. V., Shirote P. J. (2025). Recent Updates in
Nanocrystal Technology: A Reference
to Oral Dru g Delivery System. Micro Nanosyst..

[25] Pinto E. C., do Carmo F. A., Honório T. d.
S., Barros R. d. C. d. S. A., Castro H. C. R., Rodrigues C. R., Esteves V. S. A. D. A. D., Rocha H. V. A., de
Sousa V. P., Cabral L. M. (2012). Influence of the Efavirenz Micronization
on Tableting and Dissolution. Pharmaceutics.

[26] Da
Costa M., Seiceira R., Rodrigues C., Hoffmeister C., Cabral L., Rocha H. (2013). Efavirenz Dissolution
Enhancement I: Co-Micronization. Pharmaceutics.

[27] da
Costa M. A., Lione V. O. F., Rodrigues C. R., Cabral L. M., Rocha H. V. A. (2015). Efavirenz Dissolution Enhacement
II: Aqueous Co-Spray Drying. Int. J. Pharm.
Sci. Res..

[28] Hoffmeister C. R. D., da Costa M. A., Prado L. D., Rocha H. V. A., Fandaruff C., Silva M. A. S., Cabral L. M., Pitta L. R., Bilatto S. E. R., Corrêa D. S., Tasso L. (2017). Efavirenz Dissolution
Enhancement III: Colloid Milling, Pharmacokinetics and Electronic
Tongue Evaluation. Eur. J. Pharm. Sci..

[29] World Health Organization . Paediatric ARV Drug Optimization 3 Review: Summary Report; Geneva. December 12th, 2017, 2018.

[30] Sartori G. J., Prado L. D., Rocha H. V. A. (2017). Efavirenz
Dissolution Enhancement
IV—Antisolvent Nanocrystallization by Sonication, Physical
Stability, and Dissolution. AAPS PharmSciTech.

[31] Jakubowska E. (2024). A Short History
of Drug Nanocrystals–Methods, Milestones and Meaning in Pharmaceutical
Technology. J. Drug Deliv. Sci. Technol..

[32] da
Silva T. M., Honorio T. S. d. S., Chaves M. H. d. C., Cabral L. M., Patricio B. F. C. d.
C., Rocha H. V. A., Duque M. D. (2021). In Silico Bioavailability for BCS Class II Efavirenz
Tablets Using Biorelevant Dissolution Media for IVIVR and Simulation
of Formulation Changes. Drug Dev. Ind. Pharm..

[33] Wacker M. G., Lu X., Burke M., Nir L., Fahmy R. (2022). Testing the In-Vitro
Product Performance of Nanomaterial-Based Drug Products: View of the
USP Expert Panel. Dissolution Technol..

[34] Mackie C., Arora S., Seo P., Moody R., Rege B., Pepin X., Heimbach T., Tannergren C., Mitra A., Suarez-Sharp S., Borges L. N., Kijima S., Kotzagiorgis E., Malamatari M., Veerasingham S., Polli J. E., Rullo G. (2024). Physiologically Based Biopharmaceutics
Modeling (PBBM): Best Practices for Drug Product Quality, Regulatory
and Industry Perspectives: 2023 Workshop Summary Report. Mol. Pharmaceutics.

[35] Boddu R., Kollipara S., Vijaywargi G., Ahmed T. (2023). Power of Integrating
PBPK with PBBM (PBPK-BM): A Single Model Predicting Food Effect, Gender
Impact, Drug-Drug Interactions and Bioequivalence in Fasting &amp;
Fed Conditions. Xenobiotica.

[36] Yuan D., He H., Wu Y., Fan J., Cao Y. (2019). Physiologically Based
Pharmacokinetic Modeling of Nanoparticles. J.
Pharm. Sci..

[37] Nagpal S., Palaniappan T., Wang J.-W., Wacker M. G. (2024). Revisiting
Nanomedicine
Design Strategies for Follow-on Products: A Model-Informed Approach
to Optimize Performance. J. Controlled Release.

[38] Costa B., Gouveia M. J., Vale N. (2024). PBPK Modeling
of Lamotrigine and
Efavirenz during Pregnancy: Implications for Personalized Dosing and
Drug-Drug Interaction Management. Pharmaceutics.

[39] Pan X., Rowland Yeo K. (2023). Physiologically
Based Pharmacokinetic Modeling to Determine
the Impact of CYP2B6 Genotype on Efavirenz Exposure in Children, Mothers
and Breastfeeding Infants. Clin. Pharmacol.
Ther..

[40] Roberts O., Rajoli R. K. R., Back D. J., Owen A., Darin K. M., Fletcher C. V., Lamorde M., Scarsi K. K., Siccardi M. (2018). Physiologically
Based Pharmacokinetic Modelling Prediction of the Effects of Dose
Adjustment in Drug-Drug Interactions between Levonorgestrel Contraceptive
Implants and Efavirenz-Based Art. J. Antimicrob.
Chemother..

[41] Marzolini C., Rajoli R., Battegay M., Elzi L., Back D., Siccardi M. (2017). Physiologically Based Pharmacokinetic Modeling to Predict
Drug–Drug Interactions with Efavirenz Involving Simultaneous
Inducing and Inhibitory Effects on Cytochromes. Clin. Pharmacokinet..

[42] Mtshali S., Jacobs B. A. (2022). A Comparative Analysis
of Physiologically Based Pharmacokinetic
Models for Human Immunodeficiency Virus and Tuberculosis Infections. Antimicrob. Agents Chemother..

[43] Silva A. D. A., Sarcinelli M. A., Patricio B. F. d. C., Chaves M. H. d. C., Lima L. M., Parreiras P. M., Pinto P. d. F., Prado L. D., Rocha H. V. A. (2023). Pharmaceutical
Development of Micro and Nanocrystals of a Poorly Water-Soluble Drug:
Dissolution Rate Enhancement of Praziquantel. J. Drug Deliv. Sci. Technol..

[44] Mahapatra S., Thakur T. S., Joseph S., Varughese S., Desiraju G. R. (2010). New Solid State Forms of the Anti-HIV Drug Efavirenz.
Conformational Flexibility and High Z′ Issues. Cryst. Growth Des..

[45] Van de Hulst, H. C. Rigorous Scattering Theory for Spheres of Arbitrary Size (Mie Theory). In Light Scattering by Small Particles; Dover Publications Inc.: New York, NY, 1981; pp 114–130.

[46] Khan K. A. (1975). The Concept
of Dissolution Efficiency. J. Pharm. Pharmacol..

[47] Shah V. P., Tsong Y., Sathe P., Liu J.-P. (1998). In Vitro Dissolution
Profile Comparison—Statistics and Analysis of the Similarity
Factor, F2. Pharm. Res..

[48] International Council for Harmonisation of Technical Requirements for Pharmaceuticals for Human Use. M9: Biopharmaceutics Classification System-Based Biowaivers; 2019.

[49] Zhang Y., Huo M., Zhou J., Zou A., Li W., Yao C., Xie S. (2010). DDSolver: An Add-In Program for Modeling and Comparison of Drug Dissolution
Profiles. AAPS J..

[50] Prado L., de Castro I., Gonçalves K., Argenta T., Tasso L., Rocha H. V. (2025). Efavirenz
Microcrystals for Dissolution Enhancement
- Fluid Bed Granulation, Scale-up, Tableting and Pharmacokinetics. Quim. Nova.

[51] Tasso L., Barreto Argenta T., de Carvalho F. P. B., Rocha H. (2024). Rapid and Sensitive
Quantification of Efavirenz in Rat Plasma Using HPLC-MS/MS Method. Drug Anal. Res..

[52] Bergwerf, H. MolView: an attempt to get the cloud into chemistry classrooms; Commitee on Computers in Chemical Education. https://confchem.ccce.divched.org/2015FallCCCENLP9 (accessed June 6, 2025).

[53] Takano R., Sugano K., Higashida A., Hayashi Y., Machida M., Aso Y., Yamashita S. (2006). Oral Absorption
of Poorly Water-Soluble Drugs: Computer
Simulation of Fraction Absorbed in Humans from a Miniscale Dissolution
Test. Pharm. Res..

[54] Darling, I. M. ; Owen, J. S. ; Lukacova, V. Efavirenz Physiologically Based Pharmacokinetic Model Development and Validation as a Moderate CYP3A4 Inducer for Drug-Drug Interactions Predictinons. In AAPS Annu. Meet. PharmSci, 2019; Vol. 360, M1430(13–87), p 1430.

[55] Melis V., Usach I., Gandía P., Peris J. (2016). Inhibition of Efavirenz
Metabolism by Sertraline and Nortriptyline and Their Effect on Efavirenz
Plasma Concentrations. Antimicrob. Agents Chemother..

[56] Ward B. A., Gorski J. C., Jones D. R., Hall S. D., Flockhart D. A., Desta Z. (2003). The Cytochrome P450 2B6 (CYP2B6) Is the Main Catalyst of Efavirenz
Primary and Secondary Metabolism: Implication for HIV/AIDS Therapy
and Utility of Efavirenz as a Substrate Marker of CYP2B6 Catalytic
Activity. J. Pharmacol. Exp. Ther..

[57] Ahmad A., Pepin X., Aarons L., Wang Y., Darwich A. S., Wood J. M., Tannergren C., Karlsson E., Patterson C., Thörn H., Ruston L., Mattinson A., Carlert S., Berg S., Murphy D., Engman H., Laru J., Barker R., Flanagan T., Abrahamsson B., Budhdeo S., Franek F., Moir A., Hanisch G., Pathak S. M., Turner D., Jamei M., Brown J., Good D., Vaidhyanathan S., Jackson C., Nicolas O., Beilles S., Nguefack J.-F., Louit G., Henrion L., Ollier C., Boulu L., Xu C., Heimbach T., Ren X., Lin W., Nguyen-Trung A.-T., Zhang J., He H., Wu F., Bolger M. B., Mullin J. M., van Osdol B., Szeto K., Korjamo T., Pappinen S., Tuunainen J., Zhu W., Xia B., Daublain P., Wong S., Varma M. V. S., Modi S., Schäfer K. J., Schmid K., Lloyd R., Patel A., Tistaert C., Bevernage J., Nguyen M. A., Lindley D., Carr R., Rostami-Hodjegan A. (2020). IMI–Oral
Biopharmaceutics Tools Project–Evaluation of Bottom-up PBPK
Prediction Success Part 4: Prediction Accuracy and Software Comparisons
with Improved Data and Modelling Strategies. Eur. J. Pharm. Biopharm..

[58] Rajoli R. K. R. R., Back D. J., Rannard S., Freel Meyers C. L., Flexner C., Owen A., Siccardi M. (2015). Physiologically Based
Pharmacokinetic Modelling to Inform Development of Intramuscular Long-Acting
Nanoformulations for HIV. Clin. Pharmacokinet..

[59] Souček P., Gut I. (1992). Cytochromes P-450 in Rats: Structures, Functions, Properties and
Relevant Human Forms. Xenobiotica.

[60] Poulin P., Theil F. (2000). A Priori Prediction
of Tissue:Plasma Partition Coefficients of Drugs
to Facilitate the Use of Physiologically-Based Pharmacokinetic Models
in Drug Discovery. J. Pharm. Sci..

[61] Pepin X., Goetschy M., Abrahmsén-Alami S. (2022). Mechanistic
Models
for USP2 Dissolution Apparatus, Including Fluid Hydrodynamics and
Sedimentation. J. Pharm. Sci..

[62] Panikumar A. D., Venkat Raju Y., Sunitha G., Sathesh Babu P. R., Subrahmanyam C. V. S. (2012). Development of Biorelevant and Discriminating Method
for Dissolution of Efavirenz and Its Formulations. Asian J. Pharm. Clin. Res..

[63] Hammouda B. (2013). Temperature
Effect on the Nanostructureof SDS Micelles in Water. J. Res. Natl. Inst. Stand. Technol..

[64] Marques M. M., Rezende C. A., Lima G. C., Marques A. C. S., Prado L. D., Leal K. Z., Rocha H. V. A., Ferreira G. B., Resende J. A. L. C. (2017). New
Solid Forms of Efavirenz: Synthesis, Vibrational Spectroscopy and
Quantum Chemical Calculations. J. Mol. Struct..

[65] Feng T., Pinal R., Carvajal M. T. (2008). Process
Induced Disorder in Crystalline
Materials:Differentiating Defective Crystals from the Amorphous Form
of Griseofulvin. J. Pharm. Sci..

[66] Chadha R., Arora P., Saini A., Singh Jain D. (2012). An Insight
into Thermodynamic Relationship Between Polymorphic Forms of Efavirenz. J. Pharm. Pharm. Sci..

[67] de
Paiva F. C. M., Ferreira J. P. d. S., Alexandrino-Júnior F., Chaves M. H. d. C., Chrisman E. C. A. N., Sarcinelli M. A., Rocha H. V. A. (2025). From Challenge to (Dis)­Solution: Nanocrystal Technology
against the Poor Water Solubility of Lopinavir. J. Drug Deliv. Sci. Technol..

[68] Fandaruff C., Segatto Silva M. A., Galindo Bedor D. C., de Santana D. P., Rocha H. V. A., Rebuffi L., Azanza Ricardo C. L., Scardi P., Cuffini S. L. (2015). Correlation between Microstructure
and Bioequivalence in Anti-HIV Drug Efavirenz. Eur. J. Pharm. Biopharm..

[69] Csajka C., Marzolini C., Fattinger K., Decosterd L., Fellay J., Telenti A., Biollaz J., Buclin T. (2003). Population
Pharmacokinetics and Effects of Efavirenz in Patients with Human Immunodeficiency
Virus Infection. Clin. Pharmacol. Ther..

[70] Curley P., Rajoli R. K. R., Moss D. M., Liptrott N. J., Letendre S., Owen A., Siccardi M. (2017). Efavirenz Is Predicted To Accumulate
in Brain Tissue: An In Silico, In Vitro, and In Vivo Investigation. Antimicrob. Agents Chemother..

[71] U.S. Food and Drug Adminsitration . Center for Drug Evaluation and Research (CDER). In Clinical Pharmacology and Biopharmaceutics Review–NDA 20–972: Efavirenz (Sustiva); 1998.

[72] Ibarra M., Magallanes L., Lorier M., Vázquez M., Fagiolino P. (2016). Sex-by-Formulation
Interaction Assessed through a Bioequivalence
Study of Efavirenz Tablets. Eur. J. Pharm. Sci..

[73] Abhyankar D., Shedage A., Gole M., Raut P. (2017). Pharmacokinetics of
Fixed-Dose Combination of Tenofovir Disoproxil Fumarate, Lamivudine,
and Efavirenz: Results of a Randomized, Crossover, Bioequivalence
Study. Int. J. STD AIDS.

[74] Sartori G. J., Prado L. D., Rocha H. (2022). Efavirenz
Dissolution Enhancement
V–A Combined Top down/Bottom up Approach on Nanocrystals Formulation. Braz. J. Pharm. Sci..

[75] Yang H., Kim H., Jung S., Seo H., Nida S. K., Yoo S.-Y., Lee J. (2018). Pharmaceutical Strategies
for Stabilizing Drug Nanocrystals. Curr. Pharm.
Des..

[76] Azad M., Afolabi A., Bhakay A., Leonardi J., Davé R., Bilgili E. (2015). Enhanced Physical Stabilization
of Fenofibrate Nanosuspensions
via Wet Co-Milling with a Superdisintegrant and an Adsorbing Polymer. Eur. J. Pharm. Biopharm..

[77] Sun M., Guo M., He Z., Luo Y., He X., Huang C., Yuan Y., Zhao Y., Song X., Wang X. (2024). Enhanced Anti-Inflammatory
Activity of Tilianin Based on the Novel Amorphous Nanocrystals. Pharmaceuticals.

[78] Chougule M., Sirvi A., Saini V., Kashyap M., Sangamwar A. T. (2023). Enhanced
Biopharmaceutical Performance of Brick Dust Molecule Nilotinib via
Stabilized Amorphous Nanosuspension Using a Facile Acid–Base
Neutralization Approach. Drug Deliv. Transl.
Res..

[79] Martignoni M., Groothuis G. M. M., de Kanter R. (2006). Species Differences
between Mouse,
Rat, Dog, Monkey and Human CYP-Mediated Drug Metabolism, Inhibition
and Induction. Expert Opin. Drug Metab. Toxicol..

[80] Curley, P. Understanding Disposition of Efavirenz and Application in Solid Drug Nanoparticle Development; Dep. Mol. Clin. Pharmacol. Univ.: Liverpool, UK, 2015.

[81] Yau E., Olivares-Morales A., Gertz M., Parrott N., Darwich A. S., Aarons L., Ogungbenro K. (2020). Global Sensitivity Analysis of the
Rodgers and Rowland Model for Prediction of Tissue: Plasma Partitioning
Coefficients: Assessment of the Key Physiological and Physicochemical
Factors That Determine Small-Molecule Tissue Distribution. AAPS J..

[82] McDonald T. O., Giardiello M., Martin P., Siccardi M., Liptrott N. J., Smith D., Roberts P., Curley P., Schipani A., Khoo S. H., Long J., Foster A. J., Rannard S. P., Owen A. (2014). Antiretroviral Solid Drug Nanoparticles
with Enhanced Oral Bioavailability:
Production, Characterization, and In Vitro-In Vivo Correlation. Adv. Healthc. Mater..

[83] Olagunju A., Rajoli R. K. R., Atoyebi S. A., Khoo S., Owen A., Siccardi M. (2018). Physiologically-Based
Pharmacokinetic Modelling of
Infant Exposure to Efavirenz through Breastfeeding. AAS Open Res..

[84] Crommelin, D. J. A. ; de Vlieger, J. S. B. ; Mühlebach, S. Introduction: Defining the Position of Non-Biological Complex Drugs. In Non-Biological Complex Drugs; Crommelin, D. J. A. ; Vlieger, J. S. B. d. , Eds.; Springer: Cham, 2015; pp 1–8.

[85] Borchard, G. Drug Nanocrystals. In Non-Biological Complex Drugs: The Science and the Regulatory Landscape; Crommelin, D. J. A. ; Vlieger, J. S. B. d. , Eds.; Springer: Cham, 2015; pp 171–189.

[86] Singhal M., Turunen E., Ahtola-Sätilä T., Aspegren J., Bratty J. R., Fuhr R., Ojala K., van Veen B., Peltonen L. (2022). Nanoparticle-Based Oral Formulation
Can Surprise You with Inferior in Vivo Absorption in Humans. Eur. J. Pharm. Biopharm..

[87] Sreedevi A., Musmade P. B., Bhat K., Dharmagadda S., Janodia M. D., Bhat B. B., Ligade V. S. (2024). A Deep
Dive into
the Development of Complex Generics: A Comprehensive Review. J. Appl. Pharm. Sci..

[88] Au J. L.-S., Lu Z., Abbiati R. A., Wientjes M. G. (2019). Systemic Bioequivalence
Is Unlikely to Equal Target Site Bioequivalence for Nanotechnology
Oncologic Products. AAPS J..

[89] Miller N. A., Reddy M. B., Heikkinen A. T., Lukacova V., Parrott N. (2019). Physiologically
Based Pharmacokinetic Modelling for First-In-Human Predictions: An
Updated Model Building Strategy Illustrated with Challenging Industry
Case Studies. Clin. Pharmacokinet..

[90] Cristofoletti R., Rostami-Hodjegan A. (2023). Linking in
Vitro–in Vivo Extrapolations with
Physiologically Based Modeling to Inform Drug and Formulation Development. Biopharm. Drug Dispos..

[91] Gutierrez F., Navarro A., Padilla S., Anton R., Masia M., Borras J., Martin-Hidalgo A. (2005). Prediction
of Neuropsychiatric Adverse
Events Associated with Long-Term Efavirenz Therapy, Using Plasma Drug
Level Monitoring. Clin. Infect. Dis..

[92] Torno M. S., Witt M. D., Saitoh A., Fletcher C. V. (2008). Successful Use of
Reduced-Dose Efavirenz in a Patient with Human Immunodeficiency Virus
Infection: Case Report and Review of the Literature. Pharmacother. J. Hum. Pharmacol. Drug Ther..

[93] Bartlett J. A., Shao J. F. (2009). Successes, Challenges,
and Limitations of Current Antiretroviral
Therapy in Low-Income and Middle-Income Countries. Lancet Infect. Dis..

